# Gamma-Linolenic and Stearidonic Acids Are Required for Basal Immunity in *Caenorhabditis elegans* through Their Effects on p38 MAP Kinase Activity

**DOI:** 10.1371/journal.pgen.1000273

**Published:** 2008-11-21

**Authors:** Madhumitha Nandakumar, Man-Wah Tan

**Affiliations:** 1 Department of Genetics, Stanford University School of Medicine, Stanford, California, United States of America; 2 Department Microbiology and Immunology, Stanford University School of Medicine, Stanford, California, United States of America; 3 Program in Immunology, Stanford University School of Medicine, Stanford, California, United States of America; Fred Hutchinson Cancer Research Center, United States of America

## Abstract

Polyunsaturated fatty acids (PUFAs) form a class of essential micronutrients that play a vital role in development, cardiovascular health, and immunity. The influence of lipids on the immune response is both complex and diverse, with multiple studies pointing to the beneficial effects of long-chain fatty acids in immunity. However, the mechanisms through which PUFAs modulate innate immunity and the effects of PUFA deficiencies on innate immune functions remain to be clarified. Using the *Caenorhabditis elegans*–*Pseudomonas aeruginosa* host–pathogen system, we present genetic evidence that a Δ6-desaturase FAT-3, through its two 18-carbon products—gamma-linolenic acid (GLA, 18:3n6) and stearidonic acid (SDA, 18:4n3), but not the 20-carbon PUFAs arachidonic acid (AA, 20:4n6) and eicosapentaenoic acid (EPA, 20:5n3)—is required for basal innate immunity in vivo. Deficiencies in GLA and SDA result in increased susceptibility to bacterial infection, which is associated with reduced basal expression of a number of immune-specific genes—including *spp-1*, *lys-7*, and *lys-2*—that encode antimicrobial peptides. GLA and SDA are required to maintain basal activity of the p38 MAP kinase pathway, which plays important roles in protecting metazoan animals from infections and oxidative stress. Transcriptional and functional analyses of *fat-3*–regulated genes revealed that *fat-3* is required in the intestine to regulate the expression of infection- and stress-response genes, and that distinct sets of genes are specifically required for immune function and oxidative stress response. Our study thus uncovers a mechanism by which these 18-carbon PUFAs affect basal innate immune function and, consequently, the ability of an organism to defend itself against bacterial infections. The conservation of p38 MAP kinase signaling in both stress and immune responses further encourages exploring the function of GLA and SDA in humans.

## Author Summary

Polyunsaturated fatty acids are vital for optimal physiological functions, including immunity. Much of these effects are mediated by eicosanoids, which are metabolites of arachidonic acid (AA) and eicosapentaenoic acid (EPA). In mammals, PUFAs cannot be synthesized de novo. They are produced from essential dietary fatty acids, which are first converted to gamma-linolenic acid (GLA) and stearidonic acid (SDA) by a rate-limiting step catalyzed by a Δ6-desaturase, FADS2. Activity of FADS2 is impaired under numerous conditions—including aging, diabetes, stress, and smoking—and could lead to reduced production of GLA and SDA. In this study, we examined the effects of loss-of-function mutations in PUFA biosynthetic genes on the ability of *C. elegans* to survive infection by the Gram-negative human pathogen *P. aeruginosa*. We show that the enhanced pathogen susceptibility of the *C. elegans* Δ6-desaturase mutant *fat-3* is associated with decreased basal expression of immunity genes and disrupted activity of the p38 MAP kinase. These defects could be fully restored when both GLA and SDA, but not AA or EPA, were added into the diets of *fat-3* mutants, further supporting the conclusion that GLA and SDA are required for basal immunity in *C. elegans*.

## Introduction

Polyunsaturated fatty acids (PUFAs) are a class of long chain fatty acids of 18 carbon atoms or more in length that contain two or more double bonds. PUFAs are classified into two groups, the omega-6 (n-6) or the omega-3 (n-3) fatty acids, depending on the position of the double bond (n) closest to the methyl end of the fatty acid chain. In mammals, the 18-carbon and longer omega-6 and omega-3 PUFA families cannot be synthesized de novo. They are produced, instead, from the dietary essential fatty acids linoleic acid (LA, 18:2n6) and alpha-linolenic acid (ALA, 18:3n3) through a series of desaturation and elongation reactions catalyzed by desaturase and elongase enzymes, respectively [1],[2]. Omega-6 PUFAs, such as arachidonic acid (AA, 20:4n6) are converted into eicosanoids, leukotrienes and prostanoids through the actions of lipoxygenase and cyclooxygenase enzymes [3]. In vertebrates, these eicosanoids variously exert stimulatory and inhibitory influences and have profound effects on multiple aspects of organismal physiology, including immunity [4],[5]. For example, prostaglandins and leukotrienes are pro-inflammatory mediators that are vital for the initial containment of an infection and for the recruitment of phagocytes and other immune cells to a site of infection [6]. Omega-3 fatty acids such as eicosapentaenoic acid (EPA, 20:5n3) influence the T cell response to infection and demonstrate strong anti-inflammatory effects [7]. Dietary fatty acids and eicosanoids have also been shown to bind nuclear receptors, such as the Peroxisome Proliferator Activated Receptor γ (PPAR-γ), which modulates activation of dendritic cells, NK cells and T cells [8]–[10]. The influence of PUFAs on immune functions also extends to other organisms that possess only an innate immune response. For example, eicosanoids are crucial mediators and coordinators of insect cellular immune reactions to bacterial, fungal, and parasitoid invaders, specifically microaggregation, nodulation, and encapsulation [11]. In the silkworm *Bombyx mori*, eicosanoids are involved in the expression of the antibacterial proteins cecropin and lysozyme in the fat body [12]. In *Drosophila*, a functional coupling between eicosanoid biosynthesis and the IMD pathway for the induction of the antibacterial peptide diptericin by LPS has been reported [13],[14]. An analogous fatty acid-derived signaling pathway has also been shown to be important for defense in plant. However, instead of the 20-carbon AA, which is a minor PUFA in plants, 18-carbon PUFAs serve as major precursors for the synthesis of jasmonates and other oxylipins that play important roles in pathogen defense [15]. Jasmonates regulate the expression of defense genes that are essential for survival against insects and necrotrophic pathogens [16]. Oxylipins, such as crepenynic and dehydrocrepenynic acids are biologically active anti-fungal compounds [17].

Innate immunity forms a common first line of defense for most organisms, providing a highly conserved but generally non-specific response to pathogens and parasites. The innate immune system can be distinguished into two separate but overlapping components, the constitutive or basal branch of innate immune defense, and the pathogen-induced responses [18],[19]. Constitutive or basal immunity involves the constant production of effector molecules such as defensins and other antimicrobial peptides, providing a preventative barrier and allowing the organism to instantaneously respond to an immunological insult [20]–[22]. The inducible branch of the innate immune system, on the other hand, is only activated after the host has encountered a pathogen, and typically includes the induction of additional effector molecules, and where present, the recruitment and activation of phagocytic cells [23].

Both constitutive and inducible innate immunity has been described in the soil nematode *C. elegans*. For example, a number of antimicrobial peptides are constitutively expressed in healthy worms, including lysozymes [24], the ABF-2 defensin [25] and the SPP-1 saposin [24]. A subset of these constitutively-expressed antimicrobials, and a suite of additional effector molecules, including members of the C-type lectin family, are up-regulated at different time points after infection [26]–[28]. The ability of *C. elegans* to defend against infections requires several conserved signaling pathways [19],[29],[30]. They include a MAP kinase cascade, resulting in the activation of the p38 MAP kinase homologue PMK-1 [29], an insulin-like defense pathway that activates the FOXO transcription factor homologue DAF-16 [31] and a TGF-β pathway [26]. These pathways are required for the elevated production of a number of effector molecules, including antimicrobial peptides, lysozymes and lectins, to levels above those seen under basal conditions, in healthy worms [24],[28]. The p38 MAP kinase pathway also plays a vital role in maintaining the basal immune response, and mutants in this pathway, such as the p38 MAP kinase mutant *pmk-1*, show defects in the constitutive expression of lysozymes, lectins and other effector molecules [28].

**Figure 1:**
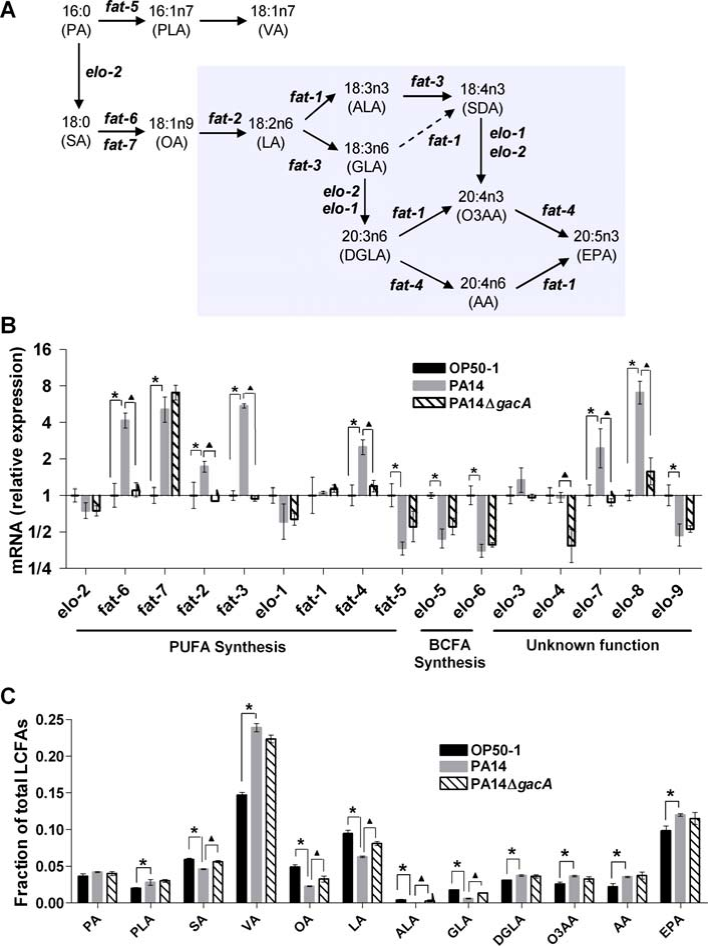
PUFA synthesis and composition are altered with *P. aeruginosa* infection. A. Schematic of the LCFA synthesis pathway in *C. elegans*, adapted from [32]. Diagram depicts all known steps in PUFA synthesis as well as the elongase and desaturase enzymes involved. Shaded section depicts synthesis of PUFAs from their monounsaturated precursors. B. qRT-PCR analysis of mRNA expression of LCFA synthesis genes in response to *P. aeruginosa* (PA14) infection. Data from three independent experiments were normalized as indicated in Materials and Methods and are depicted as mean±s.e.m., relative to age-matched animals feeding on *E. coli* OP50-1. *, p≤0.05 in the comparison between OP50-1 and PA14; ^▴^, p≤0.05 in the comparison between PA14 and PA14Δ*gacA*; Student's *t*-test. C. Relative abundance of select LCFA in PA14-infected animals. GC-MS analysis was used to measure and identify the individual fatty acid species. LCFA levels are expressed as fraction of total long chain fatty acids and are depicted as mean±s.e.m. from three independent experiments. *, p≤0.001 in the comparison between *E. coli* and PA14; ^▴^, p≤0.001 in the comparison between PA14 and PA14Δ*gacA*; Student's *t*-test. Abbreviations: AA, arachidonic acid; ALA, alpha-linolenic acid; DGLA, dihomo-γ-linolenic acid; EPA, eicosapentaenoic acid; GLA, gamma-linolenic acid; LA, linoleic acid; O3AA, ω-3 arachidonic acid; OA, oleic acid; PA, palmitic acid; PLA, palmitoleic acid; SA, stearic acid; VA, vaccenic acid.

In addition to the innate immune response, pathways for lipid synthesis and metabolism are also largely conserved in *C. elegans* [32]–[34], making the worm an ideal model to investigate the effects of lipids on immune function. Unlike mammals, *C. elegans* is able to synthesize all its required long chain fats from its bacterial food source (Figure 1A), allowing for the manipulation of lipid synthesis and content in the worm [32],[35]. Synthesis of these fatty acids is catalyzed by elongase and desaturase enzymes, which in *C. elegans* are encoded by *elo* and *fat* genes, respectively, and the *C. elegans* genome contains the full complement of enzymes required for the synthesis of long chain fatty acids (LCFAs) [32],[35]. The absence of obvious mammalian orthologs of cyclooxygenases and lipoxygenases or of prostanoid and leukotriene receptors in the *C. elegans* genome [36] provides the opportunity to investigate the roles for PUFAs in innate immunity that could otherwise be masked by the dominant influences of the prostaglandin and leukotriene eicosanoids.

Here, we use the infection of *C. elegans* by a human Gram-negative bacterial pathogen, *Pseudomonas aeruginosa* as an experimental system to investigate the interplay between PUFAs and innate immunity, in the context of the whole organism. We identify two long chain PUFAs, gamma-linolenic acid (GLA, 18:3n6) and stearidonic acid (SDA, 18:4n3), as vital for *C. elegans* defense against *P. aeruginosa* infection. Disrupting the production of these two fatty acids results in increased mortality following exposure to the pathogen. We demonstrate, by deficiency and exogenous supplementation studies, that GLA and SDA are required for both the basal activity of the p38 MAP kinase pathway and the basal expression of immunity genes.

## Results

### *P. aeruginosa* Infection Alters LCFA Synthesis and Composition

Although lipids are known to play multiple roles in immunity, relatively little evidence exists for the specific manipulation of the lipid metabolism in response to infection. Detailed analysis of a whole genome microarray study for gene expression in *P. aeruginosa*-infected *C. elegans* [27] revealed an enrichment for genes required for the synthesis of LCFAs. This modulation of the lipid metabolism in response to infection hinted at potential roles for fatty acids in *C. elegans* immunity. To confirm and extend the microarray observations, we used quantitative real time PCR (qRT-PCR) to compare mRNA levels of 16 LCFA synthesis genes in age-matched adult animals raised on *E. coli* OP50-1, the standard laboratory food source, or following infection by *P. aeruginosa* strain PA14 (Figure 1B). Since the activity of lipid metabolism genes could be greatly influenced by available nutritional sources, and having determined that the fatty acid contents of *E. coli* and *P. aeruginosa* were different (Figure S1A), we also quantified the mRNAs of *elo* and *fat* genes in worms exposed to PA14Δ*gacA*, an isogenic strain of *P. aeruginosa* PA14 in which the global virulence gene *gacA*, has been deleted [37]. PA14Δ*gacA* mutants were highly attenuated in their ability to kill *C. elegans* (Figure S1B) [38], but had a fatty acid composition very similar to the parental PA14 strain (Figure S1A), thus providing a useful control for changes due to nutritional differences between *E. coli* and *P. aeruginosa*. Of the nine *C. elegans* genes known to be involved in the synthesis of the majority of 18- and 20-carbon PUFAs and monounsaturated fatty acids (MUFAs), *fat-6*, *fat-2*, *fat-3* and *fat-4* were expressed at higher levels in worms exposed to *P. aeruginosa* than to *E. coli* or PA14Δ*gacA* (Figure 1B). These represent genes whose expressions were significantly induced under infection conditions, indicating a modulation of PUFA synthesis by *P. aeruginosa* infection. Of the five *elo* genes of unknown function, *elo-7* and *elo-8* were also significantly induced under infection conditions (Figure 1B). Although expression of *fat-5*, *elo-9* and the branched chain fatty acid (BCFA) biosynthetic genes (*elo-5* and *elo-6*) were lower in animals exposed to *P. aeruginosa* compared to *E. coli* (Figure 1B), they were also lower in animals exposed to attenuated PA14Δ*gacA*, suggesting that these changes in expression may be due to differences in fatty acid content between the bacterial species. These results confirmed a specific modulation of host LCFA synthesis in response to *P. aeruginosa* infection.

We next determined the effect of infection on the abundance of specific fatty acids in the worm. Gas chromatography followed by mass spectrometry (GC-MS) [32] was used to identify and compare the content of individual species of LCFAs in age-matched *P. aeruginosa*-infected worms and worms grown on *E. coli*. To rule out changes caused by nutritional differences between the two bacterial species, we also determined LCFA content of worms exposed to PA14Δ*gacA* mutant bacteria. We conclude that the higher levels of vaccenic acid (VA, 18:1n7) in *P. aeruginosa*-infected worms is most likely due to nutritional differences between *P. aeruginosa* and *E. coli* because the same increase was seen in worms that were exposed to the relatively avirulent PA14Δ*gacA* (Figure 1C). This is consistent with GC-MS results showing that both the *P. aeruginosa* and PA14Δ*gacA* strains had more than twice the VA content compared to *E. coli* (Figure S1A). Worms infected with *P. aeruginosa* had significantly lower levels of stearic acid (SA, 18:0), oleic acid (OA, 18:1n9), LA, ALA and GLA compared to worms exposed to *E. coli* or the attenuated PA14Δ*gacA* strains (Figure 1C). These changes in PUFA content also corresponded with the previously observed infection-induced changes in gene expression. Three of the genes up-regulated in response to infection, *fat-6*, *fat-2* and *fat-3*, are involved in the synthesis of fatty acids listed above, potentially indicating a feedback loop, where decreases in LCFA levels during infection could induce increased expression of corresponding biosynthetic genes. The infection-specific decreases in fatty acid levels led us to hypothesize that these LCFAs may be involved in immunity against pathogens.

### PUFA Composition Affects Immune Function

**Figure 2:**
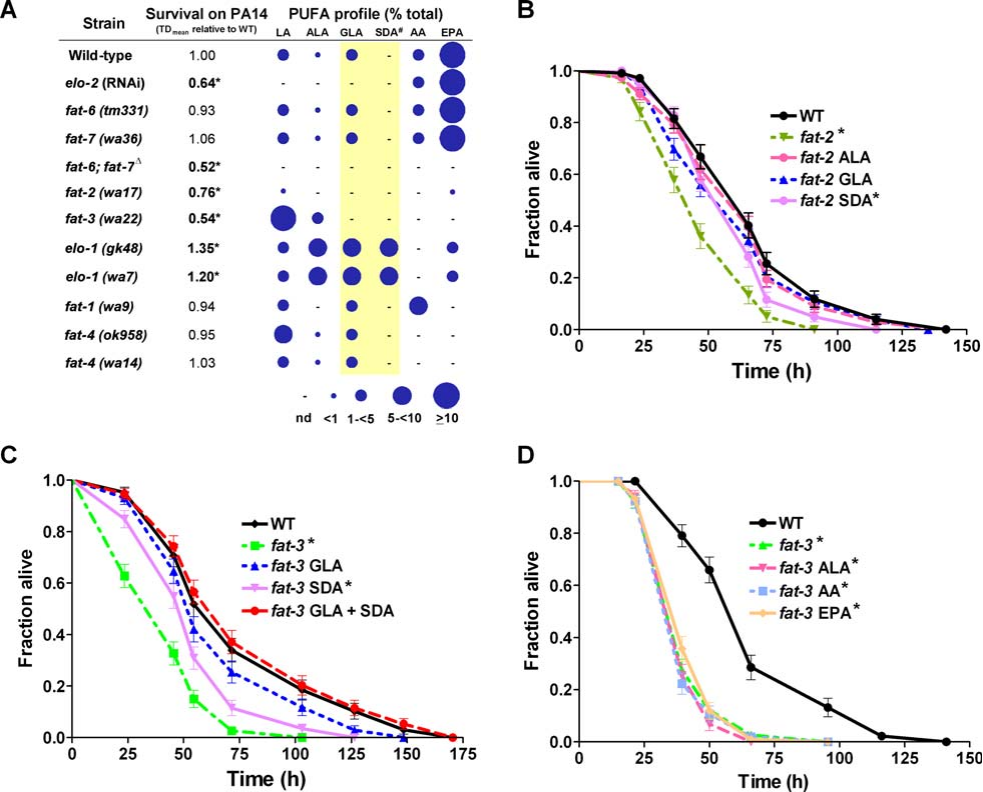
GLA and SDA are required for survival against *P. aeruginosa*. A. Fatty acid composition of PUFA synthesis mutants and their survival on *P. aeruginosa*. Mean time to death relative to wild-type (mutant TD_mean_ / wild-type TD_mean_) on *P. aeruginosa* (PA14) is shown: Values greater than 1 indicate extended survival on PA14, while values less than one indicate decreased survival relative to wild-type. Fatty acid composition as determined by GC-MS and depicted as percent of total fatty acids measured. Size of each circle represents relative amount of individual fatty acid species in the mutant. # SDA levels for *elo-1(wa7)* are as reported in [32] and for *elo-1(gk48)* are as indicated in [39], as this fatty acid was not resolvable in the GC column used in this study. Δ Fatty acid composition shown for the *fat-6(tm331)*; *fat-7(wa36)* double mutant is based on a published report [41]. B–D. Rescue of *fat-2(wa17)* and *fat-3(wa22)* survival defects by exogenous fatty acid supplementation. Age-matched adults were monitored for survival against PA14 infection. Graphs depict fraction of worms alive plotted as a function of time. Wild-type, *fat-2(wa17)* and *fat-3(wa22)* animals were supplemented with ethanol, the fatty acid solvent used in these experiments. B. Survival analysis of the *fat-2(wa17)* mutant supplemented with three different 18-carbon PUFAs. C. Survival analysis of the *fat-3(wa22)* mutant supplemented with GLA or SDA, or simultaneous supplementation with both fatty acids. D. Survival analysis of the *fat-3(wa22)* mutant supplemented with ALA, AA or EPA. *, p≤0.001 relative to wild-type within the same set of experiments; Kaplan Meier non-parametric comparison and a Logrank test. Abbreviations: AA, arachidonic acid; ALA, alpha-linolenic acid; EPA, eicosapentaenoic acid; GLA, gamma-linolenic acid; LA, linoleic acid; SDA, stearidonic acid.

To determine if specific LCFAs could be important for immune function in vivo, we analyzed a series of mutants that were unable to synthesize specific MUFAs and/or PUFAs for their ability to survive infection by *P. aeruginosa* (Figure 2A). These strong or complete loss-of-function mutants in the *fat* and *elo* genes were also analyzed by GC-MS to confirm that the genetic lesion or RNAi knockdown resulted in the expected alterations in the fatty acid profile (Figure 2A). The ELO-2 elongase is thought to catalyze the elongation of palmitic acid (PA, 16:0) to SA (Figure 1A). Reducing *elo-2* expression by RNAi resulted in significant changes in LCFA profile that is consistent with a previous report [39] and a significant increase in susceptibility to killing by *P. aeruginosa* (Figure 2A). The next step in PUFA synthesis, the conversion of SA to OA is catalyzed by two functionally-redundant desaturases, encoded by *fat-6* and *fat-7* (Figure 1A) [40],[41]. We verified previous reports that neither the loss of *fat-6* nor *fat-7* function resulted in any significant alteration in PUFA composition, and showed that neither mutant was susceptible to *P. aeruginosa* infection (Figure 2A). By contrast, the *fat-6(tm331)*; *fat-7(wa36)* double mutant, which lacked OA and the 18- and 20-carbon PUFAs derived from OA [41], was highly susceptible to killing by *P. aeruginosa*. *fat-2(wa17)* animals that lacked all 18- and most 20-carbon PUFAs were also significantly more susceptible to *P. aeruginosa* (Figure 2A). Loss of *fat-3(wa22)* function resulted in animals that lacked two specific 18-carbon PUFAs, GLA and SDA, as well as all the 20-carbon PUFAs. *fat-3(wa22)* animals were also significantly more susceptible to infection, suggesting that GLA, SDA and/or 20-carbon PUFAs that were missing in these animals could be vital for infection response in *C. elegans* (Figure 2A). Interestingly, two different *elo-1* mutants, *elo-1(gk48)* and *elo-1(wa7)*, that had decreased levels of all the 20-carbon PUFAs but accumulated the upstream 18-carbon precursors GLA and SDA [32], were significantly more resistant to killing by *P. aeruginosa* (Figure 2A). These results suggest that a lack of 20-carbon PUFAs does not compromise immune function. Consistent with this interpretation, neither the *fat-1(wa9)* mutant lacking two 20-carbon PUFAs, dihomo-γ-linolenic acid (DGLA, 20:3n6) and EPA, nor two *fat-4* mutants, *fat-4(wa14)* and *fat-4(ok958)*, that lacked AA and EPA, were significantly different from wild-type animals for susceptibility to *P. aeruginosa* (Figure 2A). Collectively, resistance of the *elo-1* mutants and increased susceptibility of the *fat-3(wa22)* mutants to *P. aeruginosa*, suggest that GLA and SDA that accumulated in the *elo-1* mutants but were absent in the *fat-3(wa22)* mutant may be required for immune function.

### Dietary Supplementation with GLA and SDA Rescues Immune Defects

PUFA levels in *fat* and *elo* mutants could be restored through dietary supplementation of the missing fatty acids [42]. To confirm the requirement of GLA and SDA for *C. elegans* to survive a pathogen challenge, *fat-2(wa17)*, *fat-3(wa22)* and *elo-1(gk48)* mutants were raised from embryos to 1-day-old adults in the presence of exogenously supplied PUFAs. A sub-population of these PUFA-supplemented adults was subjected to GC-MS to confirm that the procedure effectively restored the levels of the missing PUFAs (Figure S2) while the remaining population was subjected to survival assays. In parallel, wild-type worms were also supplemented with the respective PUFAs and the PUFA levels following supplementation were determined by GC-MS (Figure S2A). PUFA-supplemented wild-type animals were not significantly different from untreated wild-type for pathogen survival (Table 1, Figures S3A and S3B). Supplementation with ALA completely restored PUFA levels (data not shown) and survival of *fat-2(wa17)* animals on *P. aeruginosa* to that of wild-type (Figure 2B). ALA supplementation, however, failed to rescue *fat-3(wa22)* susceptibility to *P. aeruginosa* (Figure 2D, Table 1). These results were expected because the *fat-3* gene remained functional in the *fat-2(wa17)* mutant and could convert the exogenously added ALA into the required downstream fatty acids, thus rescuing the immune defects of *fat-2(wa17)* animals. The *fat-3(wa22)* mutant, on the other hand, was unable to process the supplied ALA, and consequently remained susceptible to *P. aeruginosa*. We, therefore, conclude that ALA is not directly required for immune function. Instead, ALA is likely to be modified by the FAT-3 Δ6-desaturase enzyme into functional molecules that affect immune function.

**Table 1:** PUFA supplementation rescues multiple *fat-3(wa22)* pleiotropic defects.

Strain	Supplemented PUFA	PA14 Survival (TD_mean_ relative to WT)	Lifespan (TD_mean_ (days))	Aldicarb (Mean time to paralysis (h))	Defecation pBoc intervals (s)±SD	Locomotion (body bends/min)±SD
**Wild-type**	none	1.00	12.8±0.3	16.9±1.7	48.8±3.7	206.3±12.7
	GLA+SDA	1.12	12.7±0.3	15.9±1.4	47.2±5.4	212.0±13.9
	AA	0.99	13.9±0.3*	14.8±1.3	43.6±3.5	203.0±12.5
	EPA	0.95	13.1±0.4	14.7±1.2	41.1±7.0	218.0±15.2
***fat-3(wa22)***	none	0.63*	11.5±0.3*	38.4±3.4*	63.8±8.6*	66.3±12.6*
	ALA	0.64*	11.8±0.4*	n.d.	65.2±7.3*	70.4±14.7*
	GLA	0.92	13.3±0.3	33.8±2.0*	39.3±5.8	161.3±27.7
	SDA	0.87*	13.0±0.4	37.5±4.3*	58.6±5.4*	82.2±9.3*
	GLA+SDA	1.04	12.4±0.3	30.2±3.1*	42.6±9.2	201.0±10.4
	AA	0.65*	13.3±0.3	18.3±1.9	48.7±4.9	185.3±17.8
	EPA	0.66*	13.4±0.3	16.5±1.8	42.1±7.2	172.5±21.3

Survival on *P. aeruginosa* PA14, adult lifespan, resistance to aldicarb exposure (a measure of neurotransmission), as well as defecation and locomotion of *fat-3(wa22)* and wild-type animals supplemented with dietary PUFAs compared to unsupplemented wild-type animals. PA14 survival is reported as mean time to death for each treatment relative to unsupplemented wild-type (mutant TD_mean_ / wild-type TD_mean_). Lifespan analysis is reported as mean time to death. Data from aldicarb analyses are reported as mean time to paralysis within the population tested. Statistical analyses for these three assays were performed using a Kaplan Meier non-parametric comparison and a Logrank test. For each analysis, a minimum of three independent experiments were performed, each consisting of approximately 120 worms. Analysis of defecation frequency is depicted as the average interval between successive posterior body contractions (pBoc) and data is shown as mean±SD. of the interval. Locomotion was assayed as number of body bends in liquid media over a period of two minutes [42], and depicted as average number of body bends per minute. Data for defecation and locomotion were analyzed by Student's *t*-test. In all cases, *, p≤0.001 compared to untreated wild-type worms. Abbreviations: AA, arachidonic acid; ALA, alpha-linolenic acid; EPA, eicosapentaenoic acid; GLA, gamma-linolenic acid; SDA, stearidonic acid.

Supplementation with either GLA or SDA that were absent in both the *fat-2(wa17)* and *fat-3(wa22)* mutants, resulted in a partial rescue of pathogen susceptibility (Figure 2B, C). GLA supplementation increased the mean survival period of both the *fat-2(wa17)* and *fat-3(wa22)* mutants to approximately 90% of wild-type. GLA supplementation limited to only the adult stage was also sufficient to partially rescue the susceptibility of the *fat-3(wa22)* mutant (Figure S3C), suggesting that the presence of GLA during growth and development is not necessary for its effect on the survival against *P. aeruginosa* infection. Addition of SDA alone increased the mean survival period of both *fat-2(wa17)* and *fat-3(wa22)* mutants on *P. aeruginosa* to approximately 85% of wild-type (Figure 2B, C). Supplementation with both GLA and SDA, however, completely rescued *fat-3(wa22)* survival against *P. aeruginosa* infection (Figure 2C, Table 1), indicating that GLA and SDA together are required for optimal infection response. We also note that, similar to the association between pathogen resistance and accumulation of GLA and SDA seen with *elo-1* mutants (Figure 2A), wild-type and *fat-3(wa22)* animals supplemented with both GLA and SDA were marginally more resistant to *P. aeruginosa*, although these increases were not statistically significant (Table 1).

The *P. aeruginosa*-resistant *elo-1(gk48)* animals were also supplemented with ALA, GLA and SDA. The *elo-1(gk48)* mutant already accumulated these same fatty acids (Figure 2A), and additional supplementation did not enhance pathogen resistance (Figure S3D). Pathogen resistance of *elo-1(gk48)*, coupled with wild-type phenotypes of the *fat-1(wa9)*, *fat-4(wa14)* and *fat-4(ok958)* mutants on *P. aeruginosa* (Figure 2A), indicate that 20-carbon PUFAs are not necessary for *C. elegans* immunity. To further support these conclusions, we analyzed the effect of supplementation with AA and EPA on the *fat-3(wa22)* mutant. Dietary supplementation of either AA or EPA to *fat-3(wa22)* animals effectively restored the respective PUFAs in these animals (Figure S2C, S2D), but could not rescue pathogen sensitivity of the *fat-3(wa22)* mutant (Figure 2D), confirming that the 20-carbon PUFAs do not have any detectable roles in immune function. These results further implicate the requirement for both GLA and SDA in *C. elegans* immunity.

### Intestinal PUFA Synthesis Mediated by FAT-3 Is Important for Immune Function

**Figure 3:**
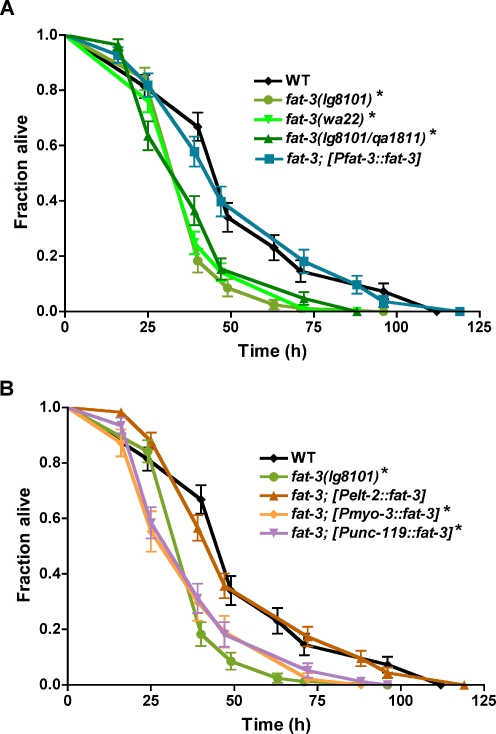
Intestinal *fat-3* expression is required for the infection response. A. *fat-3* is required for immune function. Fraction of worms alive following exposure to *P. aeruginosa*, as a function of time, for three *fat-3* alleles and a *fat-3(lg1801)* strain expressing the *fat-3* gene under its endogenous promoter (*Pfat-3::fat-3*). B. Intestinal *fat-3* expression is sufficient to rescue survival on *P. aeruginosa*. Survival analysis of *fat-3* transgenic strains expressing the *fat-3* gene under the control of tissue specific promoters. Neuronal- (*Punc-119::fat-3*) and muscle- (*Pmyo-3::fat-3*) specific transgenic rescues were in the *fat-3(lg8101)* background, while the intestine specific (*Pelt-2::fat-3*) rescue was generated in the *fat-3(lg8101/qa1811)* mutant [36]. *, p≤0.001 relative to wild-type; Kaplan Meier non-parametric analysis and a Logrank test.

The *fat-3* gene is expressed in multiple tissues, including the intestine, pharynx and body wall muscles, as well as some head and tail neurons [42]. To determine the tissue in which the *fat-3* gene is required for immune function, we obtained transgenic strains that express a functional *fat-3* gene only in the neurons, the muscles or the intestine of a *fat-3* mutant using well-established tissue-specific promoters [36] and assayed the ability of these animals to survive *P. aeruginosa* infection. We first confirmed that the two deletion alleles of *fat-3* used to generate the tissue-specific rescue strains, *fat-3(lg8101)* and *fat-3(lg8101/qa1811)* [36], demonstrated equivalent susceptibilities as *fat-3(wa22)* [32] to *P. aeruginosa* (Figure 3A). The *fat-3; [Pfat-3::fat-3]* strain, carrying a transgene consisting of the endogenous *fat-3* promoter and the *fat-3* coding region in the *fat-3(lg8101)* mutant [36], showed wild-type survival on *P. aeruginosa*, confirming that pathogen sensitivity is a direct consequence of loss of *fat-3* function (Figure 3A, Table 2). *fat-3; [Punc-119::fat-3]* transgenic animals expressing the *fat-3* coding sequence under the control of the neuron-specific *unc-119* promoter, however, remained significantly more susceptible to *P. aeruginosa* (Figure 3B, Table 2). Expression of *fat-3* under the control of the muscle-specific *myo-3* promoter also failed to rescue the *fat-3* mutant sensitivity to *P. aeruginosa* (Figure 3B, Table 2). Together, these results indicate that *fat-3* gene expression and *fat-3*-dependent PUFA synthesis in the muscles or neurons does not significantly affect immune function. By contrast, *fat-3; [Pelt-2::fat-3]* transgenic animals expressing the *fat-3* gene under the control of the intestine-specific *elt-2* promoter showed survival kinetics on *P. aeruginosa* that were indistinguishable from wild-type (Figure 3B, Table 2). Intestine-specific rescue of *fat-3* pathogen sensitivity indicates that *fat-3*-dependent synthesis of PUFAs in the intestine is sufficient for normal immune function in response to bacterial infection.

**Table 2:** Analysis of tissue-specific *fat-3* gene expression on infection survival, defecation and locomotion.

Strain	Transgene expression	Survival on PA14 (TDmean relative to WT)	Defecation (pBoc intervals (s))±SD	Locomotion (body bends/min)±SD
**Wild-type**	-	1.00	48.4±3.6	231.4±18.2
***fat-3 (lg8101)***	-	0.74*	69.8±4.4*	62.8±13.9*
***fat-3; [Pelt-2::fat-3]***	Intestine	0.97	51.7±6.9	122.4±15.2*
***fat-3; [Pmyo-3::fat-3]***	Muscles	0.76*	65.4±11.8*	71.9±10.4*
***fat-3; [Punc-119::fat-3]***	Neurons	0.81*	49.8±3.4	174.1±33.8
***fat-3; [Pfat-3::fat-3]***	All^#^	1.09	45.7±6.0	193.9±21.0

Effects of tissue-specific *fat-3* gene expression in the *fat-3(lg8101)* mutant on infection survival, defecation rate and locomotion compared to wild-type. Assays and statistical analyses were performed as described in Table 1. In all cases, *, p≤0.001 compared to wild-type. # refers to all the tissues in which *fat-3* is normally expressed because the *fat-3; [Pfat-3::fat-3]* strain expresses *fat-3* under the control of its endogenous promoter.

### The *fat-3* Mutant Neuromuscular Defects and Stress Sensitivities Can Be Dissociated from Immune Defects

Animals that have lost *fat-3* gene function display pleiotropic abnormalities, including impaired motility, a weakened cuticle, decreased defecation rate and irregular expulsion [36],[42]. Many of these defects are associated with impaired neurotransmission due the loss of *fat-3* function in the neurons [36],[42]. We also found that *fat-3(wa22)* animals had a marginal but significant decrease in adult lifespan (Table 1), contrary to a previous report [42]. Together, these defects may indicate a general poor health of *fat-3* mutants that could indirectly impact their ability to survive *P. aeruginosa* infection. To determine if these pleiotropies could be dissociated from immune defects, we first analyzed the effects of PUFA supplementation in *fat-3(wa22)* animals on these defects, in addition to survival on *P. aeruginosa*. We note that, with the possible exception of AA on adult life span, PUFA supplementations did not have any significant effects on wild-type animals (Table 1). Consistent with a previous report, supplementation with GLA [42] or GLA and SDA combined rescued the defecation and locomotion defects, and lifespan of *fat-3(wa22)* animals (Table 1). GLA and SDA, however, failed to rescue aldicarb resistance indicating that these PUFAs were not sufficient to restore synaptic transmission, as measured by acetylcholine release [43] (Table 1). By contrast, supplementation with either of the 20-carbon PUFAs, AA or EPA rescued *fat-3(wa22)* for all the phenotypes tested: adult lifespan, aldicarb resistance, defecation and locomotion defects (Table 1). Yet, *fat-3(wa22)* animals supplemented with either EPA or AA remained sensitive to *P. aeruginosa* (Figure 2D, Table 1). Failure to rescue the *fat-3(wa22)* immune defect was not due to insufficient incorporation of EPA or AA because the levels of these 20-carbon PUFAs in the *fat-3(wa22)* mutants following supplementation was equivalent to, or higher than, in wild-type (Figure S2C and S2D). Since AA or EPA could rescue *fat-3(wa22)* neuronal and muscular defects and adult lifespan but not pathogen sensitivity, while GLA and SDA rescued pathogen sensitivity, but not neurotransmission (Figure 2C and 2D, Table 1), we can conclude that pathogen susceptibility of the *fat-3(wa22)* mutant was not due to neuromuscular defects or a shortened adult lifespan. Instead, pathogen sensitivity of *fat-3* mutants is likely to be caused by factors dependent on levels of GLA and SDA.

This conclusion is further supported by tissue-specific rescue experiments using transgenic animals. Intestinal expression of the *fat-3* gene only partially rescued the locomotion defects (Table 2) despite completely rescuing the pathogen sensitivity of the *fat-3(lg8101)* mutant (Figure 3B, Table 2). Full rescue of the defecation defect and the partial rescue of locomotion by intestinal expression of *fat-3* are not surprising because most FAT-3 protein is in the intestine [42] and it is therefore likely that PUFAs synthesized in the intestine could be transported to other parts of the body. As with pathogen sensitivity, muscle-specific expression of *fat-3* was not sufficient to rescue defecation and movement defects (Table 2). By contrast, neuronal expression of the *fat-3* gene that also failed to rescue pathogen susceptibility (Figure 3B), could fully rescue the defecation and locomotion defects of the *fat-3(lg8101)* mutant (Table 2), indicating neuromuscular and immune functions may be independently regulated by *fat-3*. Together, the PUFA supplementation and tissue-specific rescue experiments indicate that the susceptibility of *fat-3* mutants to *P. aeruginosa* infection is not associated with neuromuscular and lifespan defects.

To further rule out the possibility that the increased pathogen sensitivity of *fat-3(wa22)* mutants was a consequence of a general increased sensitivity to any insults, we determined the ability of *fat-3(wa22)* animals to survive or develop under a number of additional stress conditions. We assayed the sensitivity of *fat-3* animals to heavy metal stresses by determining the proportion of embryos that could develop into adults in the presence of toxic concentrations of cadmium or copper metals [44]. Exposure to toxic levels of cadmium results in cell damage and is thought to induce the transcription of a number of defense and repair genes [45]–[47]. Following exposure to 30 µM cadmium chloride, less than 65% of *fat-3(wa22)* embryos successfully developed into adults. By contrast, approximately 75% of wild-type embryos grew to adults, indicating that *fat-3(wa22)* animals were more sensitive to cadmium (Table 3). *fat-3(wa22)* animals were also more sensitive to copper, with significantly fewer *fat-3(wa22)* adults than wild-type developed from embryos following exposure to 250 µM copper sulfate (Table 3). The *fat-3(wa22)* mutant was also more susceptible to a 1% solution of the detergent Triton X-100. Approximately 26% of *fat-3(wa22)* animals survived a 1-hour incubation with the detergent, compared to almost 83% for wild-type (Table 3). This susceptibility may be associated with the compromised cuticle of the *fat-3* mutant [42], but may also indicate defects in membrane structure and permeability in *fat-3(wa22)* animals due to the absence of long chain unsaturated fatty acids. Supplementation with GLA and SDA, as well as AA or EPA fully rescued *fat-3(wa22)* susceptibility to both heavy metals and to detergent (Table 3). This may indicate that susceptibility to these stresses is not specific to a particular PUFA species but dependent, instead, on the total level of unsaturated fatty acids in the animal. Importantly, that AA or EPA rescued *fat-3(wa22)* susceptibility to these abiotic stresses but not susceptibility to pathogens further dissociated immune function from general stress resistance in the *fat-3(wa22)* mutant.

**Table 3:** *fat-3(wa22)* mutants are sensitive to heavy metal and detergent but not to extreme temperatures.

Strain	Supplemented PUFA	Thermal stress (% alive±s.e.m.)		Heavy metals (% adults±s.e.m)		Detergent (% alive±s.e.m)
		36°C (10 h)	0°C (24 h)	CuSO_4_ (250 µM)	CdCl_2_ (30 µM)	Triton X-100 (1%)
**Wild-type**	**none**	60.4±3.4	67.0±1.6	79.1±1.5	74.8±2.1	82.8±0.5
***fat-3(wa22)***	**none**	96.1±1.7*	87.8±1.0*	67.7±0.9*	63.6±0.7*	26.5±2.3*
	**GLA+SDA**	90.8±2.0*	80.2±0.4*	84.8±1.4	79.7±2.0	74.8±3.6
	**AA**	72.4±1.4	86.0±1.1*	75.7±2.2	73.3±1.5	85.6±3.9
	**EPA**	61.9±1.2	83.2±1.1*	77.7±2.0	73.3±1.5	75.6±4.4
***sek-1(km4)***	**none**	63.9±0.5	65.0±1.7	55.0±1.4*	66.4±0.2*	83.0±3.0

The effects of mutations and PUFA supplementations on *C. elegans* sensitivity to abiotic stresses are shown. Sensitivities to extreme temperatures are depicted as percent of animals alive after the indicated time of exposure to 36°C or 0°C. Metal toxicity results are depicted as the percent of embryos that successfully developed into adults in the presence of the indicated concentrations of copper and cadmium metals. Sensitivity to detergent is shown as percent of worms alive after a 1-h exposure to 1% Triton X-100 solution. *, p≤0.001 compared to wild-type animals; Student's *t*-test. Abbreviations: AA, arachidonic acid; EPA, eicosapentaenoic acid; GLA, gamma-linolenic acid; SDA, stearidonic acid.

The physiological temperature that supports *C. elegans* development ranges from 15–25°C [48]. To determine the ability of *fat-3(wa22)* animals to tolerate extreme temperatures, we assayed for the number of one-day old adults that remained alive following exposure to 36°C and 0°C for a defined period. In contrast to heavy metal and detergent stresses, *fat-3(wa22)* animals were more resistant than wild-type to extreme temperatures. A significantly higher proportion of *fat-3(wa22 )* than wild-type animals survived the 36°C heat stress for 10 hours (Table 3) and 12 hours (data not shown). Similarly, following a 24-hour exposure to 0°C cold stress, significantly more *fat-3(wa22)* than wild-type adults remained alive (Table 3). The findings that supplementation with GLA and SDA did not restore cold and heat resistance (Table 3), but effectively restored pathogen sensitivity of the *fat-3(wa22)* mutant to wild-type (Table 1) further disassociates resistance to extreme temperatures from pathogen susceptibility. The observations that *fat-3(wa22)* animals were not always more sensitive than wild-type to all the abiotic insults tested, and that heavy metal and detergent sensitivity but not immune functions could be rescued by AA or EPA, further support the hypothesis that susceptibility of the *fat-3(wa22)* animals to *P. aeruginosa* is likely to be due to specific immune defects.

### Immune Gene Expression Is Misregulated in the *fat-3* Mutant

**Figure 4:**
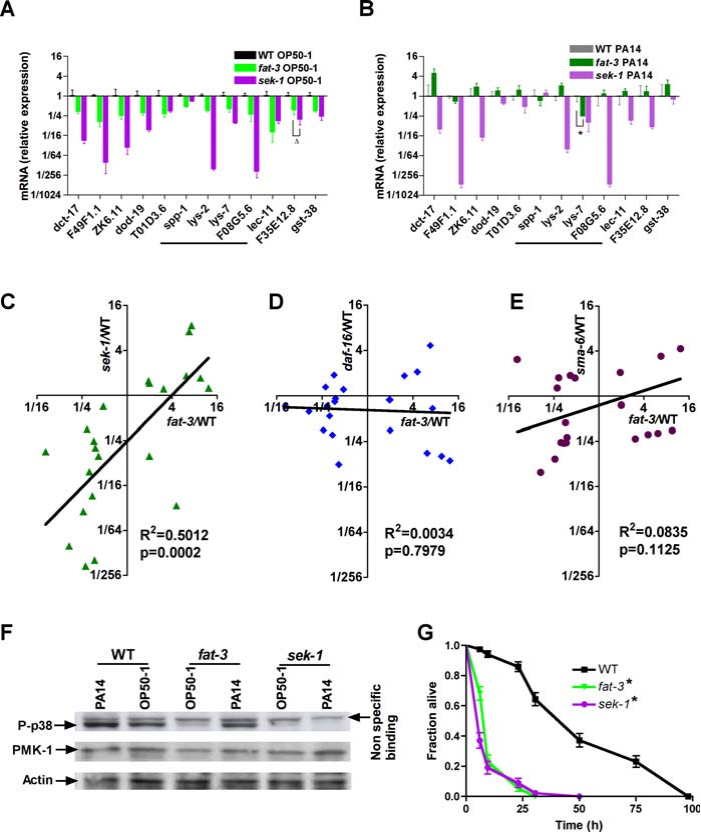
Defective basal immune gene expression and p38 MAP kinase activation in the *fat-3(wa22)* mutant. Expression of 12 infection- and stress-response genes under normal growth (A) and infection (B) conditions. mRNA expression was quantified by qRT-PCR in wild-type, *fat-3(wa22)* and *sek-1(km4)* 1-day-old adults exposed to OP50-1 (A) or PA14 (B) for 12 h. Data are depicted as mean±s.e.m and represent fold expression in mutant animals relative to wild-type under the same conditions, with wild-type set to one. A. Δ, p>0.05 between the *fat-3(wa22)* and *sek-1(km4)* mutants; Student's *t*-test. B. *, p<0.05 between *fat-3(wa22)* and wild-type; Student's *t*-test. C–E. Regression analyses of gene expression in uninfected *fat-3(wa22)* animals compared to null alleles of *sek-1(km4)* (C), *daf-16(mu86)* (D) and *sma-6(wk7)* (E). Analysis included 22 genes with significantly altered basal expression in the *fat-3(wa22)* mutant relative to wild-type (p≤0.05). Data were compared using Pearson linear regression analysis with best fit and t-tests for statistical analyses. Points represent fold difference in gene expression relative to wild-type animals. F. *fat-3(wa22)* animals show decreased basal PMK-1 phosphorylation. Immunoblot analysis of PMK-1 activation in uninfected (OP50-1) and infected (PA14) *fat-3(wa22)* worms compared to wild-type and *sek-1(km4)* animals. Lysates from age-matched young adult animals exposed to OP50-1 or PA14 for a period of 12 h were analyzed using a monoclonal antibody specific to the doubly phosphorylated activated form of the p38 protein. Arrow on right indicates an additional non-specific band seen with the phospho-p38 specific antibody. Anti-actin antibody was used as loading control. G. Survival analysis of animals exposed to arsenic-induced oxidative stress. One-day-old adult wild-type, *sek-1(km4)* and *fat-3(wa22)* animals were placed on plates coated with a final concentration of 3 mM arsenic and survival was monitored every 12 h. Graph depicts fraction of worms alive plotted as a function of time. *, p≤0.001 relative to wild-type; Kaplan Meier non-parametric comparison and a Logrank test.

The requirement for *fat-3* in the intestine, the primary site of *P. aeruginosa* infection in *C. elegans*, raised the possibility that GLA and SDA could influence immune gene expression. To provide a further link between *fat-3* gene function and innate immunity, we compared the expression of 50 infection-response genes [49] by qRT-PCR, in 1-day-old adult wild-type and *fat-3(wa22)* animals (Table S1). These 50 genes were selected based on one or more of the following criteria: a) genes with known or predicted antimicrobial activity, including *spp-1* [50] and *abf-2* [25], b) genes required for survival against *P. aeruginosa* infection [27],[28], and c) genes known to be differentially regulated in response to *P. aeruginosa* infection [27],[28]. We quantified the expression of these genes under normal growth conditions on *E. coli* to determine basal or constitutive mRNA levels, and following a 12-hour exposure to *P. aeruginosa* to compare mRNA levels in infected animals (Table S1). The constitutive expression of 22 genes (44%) was significantly different between age-matched *fat-3(wa22)* and wild-type animals raised on *E. coli*. Of these, 12 genes were expressed at significantly lower levels (Figure 4A) and 10 were expressed at significantly higher levels (Table S1) in the *fat-3(wa22)* mutant compared to wild-type. These results indicated that the missing PUFAs in the *fat-3(wa22)* mutant are required for proper basal or constitutive expression of a significant subset of infection-response genes tested. Genes expressed at lower levels in *fat-3(wa22)* included three antimicrobial peptide homologs, *spp-1*, encoding a saposin-like protein, and two lysozymes, encoded by *lys-2* and *lys-7* (Figure 4A). The reduced expression of these putative antimicrobial genes in uninfected animals raised the possibility that basal immune function of the *fat-3(wa22)* mutant may be compromised. To address this hypothesis, we first determined if the 12 constitutively down-regulated genes were required for immunity in wild-type animals. We found that RNAi-mediated knockdown of *spp-1*, *lys-2*, *lys-7*, *dct-17* and F08G5.6 resulted in significantly increased sensitivity to *P. aeruginosa*-mediated killing (Table 4). The remaining genes that did not induce any survival defects on *P. aeruginosa* following RNAi knockdown were further tested using a colonization assay. Comparing the degree of intestinal colonization by PA14-GFP, a derivative of *P. aeruginosa* PA14 that expresses the GFP protein [38], provides a more sensitive measure of the infection process, allowing us to detect smaller defects in worm immunity. RNAi-mediated knockdown of *lec-11* and F49F1.1 resulted in a significant increase in the rate of colonization by PA14-GFP (Figure S4A), despite a wild-type survival phenotype on the pathogen (Table 4). We henceforth refer to these seven infection-response genes as immunity genes, due to their role in protecting *C. elegans* from infection. The demonstration that a majority of the genes that were expressed at reduced levels in *fat-3(wa22)* animals were functionally important for immunity against *P. aeruginosa* provides a potential molecular basis for the sensitivity of the *fat-3(wa22)* mutant to infection and suggests that the absence of GLA and SDA can compromise basal immunity.

**Table 4:** Effect of gene inactivation by RNAi on sensitivity to pathogen infection and arsenic stress.

Group	Gene inactivated by RNAi	Sequence ID	Description	Mean time to death relative to control	
				Survival on *P. aeruginosa*	Survival on arsenic
	Control		Empty vector control	1.00	1.00
	*sek-1*	R03G5.2	Mitogen-activated protein kinase	0.80*	0.76*
**1**	*dct-17*	F35E12.7	CUB-like domain protein	0.80*	0.81*
	F49F1.1	F49F1.1	Metridin-like ShK toxin	0.89^#^	0.79*
**2**	ZK6.11	ZK6.11	DUF-274 domain protein	1.00	0.93
	*dod-19*	ZK6.10	DUF-274 domain protein	0.91	0.90
	T01D3.6	T01D3.6	von Willebrand factor and related coagulation proteins	1.01	0.98
**3**	*spp-1*	T07C4.4	saposin	0.85*	0.87
	*lys-2*	Y22F5A.5	N-acetylmuraminidase/ lysozyme	0.86*	1.02
	*lys-7*	C02A12.4	N-acetyluuraminidase/ lysozyme	0.85*	0.97
	F08G5.6	F08G5.6	CUB-like domain protein	0.82*	0.99
	*lec-11*	F38A5.3	Galactin, Galactose-binding lectin	0.99^#^	0.91
**4**	F35E12.8	F35E12.8	CUB-like domain protein	0.89	0.82*
	*gst-38*	F35E8.8	Glutathione S-transferase	0.97	0.76*

Values represent survival of RNAi-treated animals challenged with *P. aeruginosa* infection or arsenic-mediated oxidative stress. Survival analysis is depicted as mean time to death relative to control animals treated with a vector construct with no RNAi target (mutant TD_mean_ / control TD_mean_). *, p≤0.001 compared to control; Kaplan Meier non-parametric comparisons and a Logrank test. #, genes that, when inactivated by RNAi, did not significantly affect survival on *P. aeruginosa* but were colonized by *P. aeruginosa* to a significantly higher degree (see Figure S4A).

We next compared expression levels of the 50 infection-response genes following a 12-hour infection with *P. aeruginosa*. The mRNA levels of only seven genes (14%) were significantly different between *P. aeruginosa*-infected *fat-3(wa22)* and wild-type animals (Figure S4B, Table S1), indicating that majority of the genes in the *fat-3(wa22)* mutant, including a number of genes misregulated under basal conditions, responded to *P. aeruginosa* infection to reach levels similar to the wild-type worm. With the exception of *lys-7*, the mRNA levels of all the genes that were constitutively expressed at lower levels in uninfected *fat-3(wa22)* animals were indistinguishable from wild-type following *P. aeruginosa* infection (Figure 4B, Table S1), indicating that, at the level of gene expression, the ability of *fat-3(wa22)* mutants to respond to infection remained largely intact. Taken together, these results indicate that despite displaying a largely normal inducible response to infection, the significant reduction in constitutive expression of immunity genes was sufficient to render *fat-3(wa22)* animals more susceptible to *P. aeruginosa*-mediated death. These results underscore the importance of basal or constitutive immunity for protection from pathogens.

Given that *fat-3* expression in the intestine is required to protect *C. elegans* from *P. aeruginosa*-mediated killing (Figure 3B), we wondered if expressing *fat-3* in the intestine would be sufficient to restore the expression of infection-response genes in the *fat-3* mutants. Infection-response gene expression was quantified by qRT-PCR in transgenic *fat-3* strains that specifically express the *fat-3* transgene in the intestine, muscles or neurons. As these transgenic strains were constructed in the *fat-3(lg8101)* background, we first confirmed that, with the exception of *lec-11*, the basal gene expression of the 12 genes assayed were similarly misregulated in *fat-3(lg8101)* and *fat-3(wa22)* relative to wild-type (Table S2). The reason for this allele-specific effect on *lec-11* expression is currently unclear. Excluding *lec-11* from the remaining analysis with transgenic animals, we note that the expression of the *fat-3* gene under the control of its own promoter was sufficient to rescue the expression of all but one of the 11 infection-response genes tested (Figure S5A), indicating that the requirement for *fat-3* in immune function is strongly correlated with the expression of infection-response genes. Intestine-specific expression of *fat-3* restored the expression of seven of the eight the down-regulated infection-response genes, including the immune-specific genes *spp-1*, *lys-7* and F08G5.6 (Figure S5B), indicating that *fat-3* is required in the intestine to regulate basal gene expression. By contrast, and consistent with the pathogen survival assay (Figure 3B), expression of *fat-3* specifically in the muscles failed to restore the expression of any of the genes tested, with the exception of F35E12.8 (Figure S5C). Expression of *fat-3* in neuronal tissues was similarly ineffective at restoring infection-response gene expression; expression of only two genes, F35E12.8, and F08G5.6 were restored to wild-type (Figure S5D). Analysis of *spp-1* expression in these transgenic animals is revealing, as *spp-1* is expressed only in the intestine [24]. Expression of *spp-1* was restored to wild-type levels when *fat-3* was expressed specifically in the intestine (Figure S5B) but not in the muscles (Figure S5C) or neurons (Figure S5D). Together, these data confirm our hypothesis that *fat-3* functions in the intestine to influence the expression of a number of infection-response genes that contribute to the protecting *C. elegans* from *P. aeruginosa* infection.

### Basal p38 MAP Kinase Pathway Activity Is Compromised in *fat-3* Mutants

Of the 12 genes that are positively regulated by *fat-3* (Figure 4A), the expression of *lys-2*, *dod-19*, ZK6.11 and F08G5.6 has been reported to be dependent on the p38 MAP kinase pathway [28]. To determine if altered constitutive gene expression in the *fat-3(wa22)* mutant correlated with defects in p38 MAP kinase signaling, we compared mRNA levels between *fat-3(wa22)* and *sek-1(km4)*, a p38 MAP kinase kinase mutant [29], adults raised on *E. coli*. Using the set of 22 genes that were significantly altered in *fat-3(wa22)* animals, we found that basal gene expression between *fat-3(wa22)* and *sek-1(km4)* animals was highly correlated (Figure 4C), with the expression of 18 out of 22 genes being similarly altered in both strains. A majority of these genes, however, were expressed at lower levels in *sek-1(km4)* compared to *fat-3(wa22)* animals (Figure 4A and C). A similarly significant correlation was seen between *fat-3(wa22)* animals and another MAP kinase pathway mutant, the p38 MAP kinase homolog, *pmk-1(km25)* (R^2^ = 0.4409, p = 0.0008). Significant correlations in basal gene expression were also seen between *fat-3(wa22)* and *sek-1(km4)* (R^2^ = 0.332, p = 0.0001), and between *fat-3(wa22)* and *pmk-1(km25)* (R^2^ = 0.259, p = 0.0003) animals when the analysis was extended to the entire 50 gene-set.

In addition to the p38 MAP kinase pathway, the Sma/TGF-beta and Insulin/Insulin growth factor signal transduction pathways also play important roles in *C. elegans* immunity [19],[51],[52]. However, basal gene expression in *fat-3(wa22)* animals was not significantly correlated with the null allele of the FOXO transcription factor of the insulin pathway, *daf-16(mu86)* (Figure 4D), or the null allele of the Sma/TGF-beta receptor, *sma-6(wk7)* (Figure 4E). This high concordance in altered basal gene expression between *fat-3(wa22)* and the *sek-1(km4)* or *pmk-1(km25)* mutants led us to hypothesize that the basal activity of the p38 MAP kinase pathway may be compromised in *fat-3(wa22)* animals.

As a direct measure of the effect of the *fat-3* mutation on p38 MAP kinase pathway activity, we used immunoblot analyses to determine the levels of activated PMK-1 protein in *fat-3(wa22)* and wild-type age-matched adults raised on *E. coli*. The *sek-1(km4)* mutant, previously shown to have a complete loss of PMK-1 phosphorylation and increased susceptibility to infection [29], was used as a control. Wild-type worms had detectable levels of phosphorylated PMK-1 indicating some basal p38 MAP kinase activity under normal physiological conditions. By contrast, *fat-3(wa22)* had decreased levels of phosphorylated PMK-1 protein (Figure 4F), indicating a reduction in basal activity of the p38 MAP kinase pathway. This decrease in the basal levels of activated PMK-1 in *fat-3(wa22)* animals, as opposed to the complete loss of phosphorylated PMK-1 protein in *sek-1(km4)* animals, is consistent with the trends indicated by the qRT-PCR analysis, showing that the expression levels of infection-response genes in the *fat-3(wa22)* mutant were not as low as in the *sek-1(km4)* mutant (Figure 4A). The decrease in PMK-1 phosphorylation and immune gene expression indicate that although the *fat-3(wa22)* null mutation does not completely abolish PMK-1 activity, it is sufficient to compromise immune function in the worm.

As shown in Figure 4B and Table S1, a majority of the infection-response genes were expressed at wild-type levels in infected *fat-3(wa22)* animals. Consistent with this gene expression data, immunoblot analysis of PMK-1 activation in *fat-3(wa22)* and wild-type lysates following a 12-hour exposure to *P. aeruginosa* revealed that PMK-1 phosphorylation in the infected *fat-3(wa22)* mutant was restored to 81% of that seen in infected wild-type animals (Figure 4F). By contrast, in *sek-1(km4)* animals, the level of phosphorylated PMK-1 protein remained at background following infection, (Figure 4F), and immunity genes that were expressed at low levels under basal condition remained low following PA14 infection (Figure 4B). Thus, both the gene expression and PMK-1 phosphorylation analyses support the conclusion that FAT-3 Δ6-desaturase is necessary to maintain basal activation of PMK-1 but appears to be dispensable for PMK-1 activation and the associated immune gene expression during infection.

Since the loss of *fat-3* gene function resulted in the reduced phosphorylation of PMK-1, *fat-3(wa22)* mutants are expected to manifest phenotypes that are associated with a loss or reduction in p38 MAP kinase signaling. A well-characterized defect of the *sek-1(km4)* mutant that is associated with a complete loss of PMK-1 phosporylation is an increased susceptibility to arsenic-induced oxidative stress [53]. We therefore compared the ability of 1-day-old adult *sek-1(km4)* and *fat-3(wa22)* mutants to survive on 3 mM arsenic. As expected, *fat-3(wa22)* and *sek-1(km4)* animals had similar survival rate following exposure to arsenic (Figure 4G), further indicating that the p38 MAP kinase pathway is functionally compromised in *fat-3(wa22)* animals.

The increased sensitivity of *fat-3* loss-of-function mutants to pathogen infection and arsenic stress is associated with reduced basal p38 MAP kinase signaling. Among the genes that positively regulated by *fat-3* are three antimicrobial peptide homologs: *spp-1*, which encodes a saposin-like protein, and *lys-2* and *lys-7* that are predicted to encode for lysozymes (Figure 4A). This raises the possibility that the influence of *fat-3* on immune function may be distinct and separable from its arsenic-induced oxidative stress response. To identify *fat-3*-regulated genes that are specifically require for immunity, we inactivated each of the 12 genes down-regulated in the *fat-3(wa22)* mutant individually by RNAi and determined the effects of gene knockdown on pathogen and arsenic sensitivity. As shown in Table 4, four groups of genes were identified. Members of the first group, *dct-17* and F49F1.1, were like *sek-1(km4)* and *fat-3(wa22)* in that they were required to protect *C. elegans* from both pathogen and arsenic. By contrast, inactivation of the group 2 genes ZK6.11, *dod-19* and T01D3.6, had no detectable effect on pathogen or arsenic survival. Of particular interest are members of group 3, *spp-1*, *lys-2* and *lys-7*, F08G5.6 and *lec-11*, that are specific to immune functions; they were required to protect *C. elegans* from *P. aeruginosa* infection but not from arsenic-induced oxidative stress. F35E12.8 and *gst-38* are members of group 4 that were not required for *C. elegans* survival against infection, but were required for the response to oxidative stress. Interestingly, *gst-38* is predicted to encode a glutathione-S-transferase that plays an important role in protection from oxidative stress. We are thus able to distinguish among the *fat-3*-regulated genes, a set of immune-specific genes that are distinct from those required for protection from arsenic toxicity. These results strongly indicate that *fat-3* influences the expression of genes that have specific role in innate immunity.

### Supplementation with GLA and SDA Rescues Basal PMK-1 Phosphorylation

**Figure 5:**
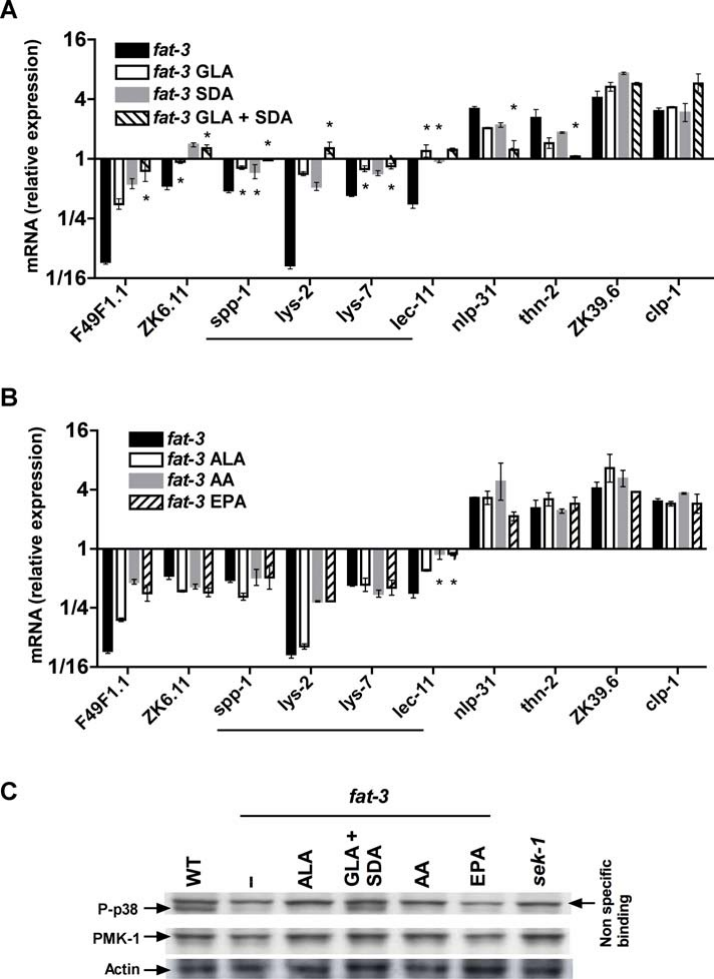
Supplementation with GLA and SDA rescues immune gene expression and PMK-1 phosphorylation. A, B. qRT-PCR analysis of basal immune gene expression in PUFA supplemented *fat-3(wa22)* animals. Exogenous supplementation with GLA and SDA rescues immune gene expression (A), while supplementation with 20-carbon PUFAs does not (B). Data are depicted as mean±s.e.m and represent fold difference in expression relative to wild-type animals on OP50-1. Significance was determined using the student's t-test. *, gene expression not significantly different (p>0.05) from wild-type, which is set at 1; Student's *t*-test. Horizontal lines in panels A and B identify genes that are specifically required for survival against PA14 infection (see Table 4). C. Immunoblot analysis of PMK-1 phosphorylation in *fat-3(wa22)* animals supplemented with 18- and 20-carbon PUFAs. Arrow on right indicates additional non-specific band seen with the phospho-p38 specific antibody. Abbreviations: AA, arachidonic acid; ALA, alpha-linolenic acid; EPA, eicosapentaenoic acid; GLA, gamma-linolenic acid; SDA, stearidonic acid.

The specific rescue of *fat-3(wa22)* survival on *P. aeruginosa* by GLA and SDA led us to hypothesize that basal PMK-1 phosphorylation and expression of infection-response genes would be restored with the supplementation of these PUFAs. As with the pathogen survival assay, we determined the effect of PUFA supplementations on the basal expression of 10 infection-response genes, 6 that were down-regulated and 4 that were significantly up-regulated in the *fat-3(wa22)* mutant, by qRT-PCR (Figure 5). As expected, individual addition of GLA or SDA fatty acids resulted in a partial restoration of immune gene expression in uninfected *fat-3(wa22)* worms, while simultaneous addition of both fatty acids completely restored the majority of basal immune gene expression in the *fat-3(wa22)* mutant to wild-type levels (Figure 5A), including *spp-1*, *lys-2*, *lys-7* and *lec-11* that are specifically required for immune function (Table 4). By contrast, supplementation with ALA, AA or EPA, the PUFAs that did not rescue *fat-3(wa22)* pathogen sensitivity (Figure 2D), also had no significant effect on the expression of these misregulated infection-response genes, with the exception of *lec-11* (Figure 5B). Two genes that had no effect on survival when knocked down by RNAi; *clp-1* and ZK39.6 (data not shown), also did not appear to be affected by the addition of any of the above PUFAs.

The conclusion that both GLA and SDA are specifically required for survival against PA14 infection, through the regulated expression of infection response genes, was further supported by immunoblot assays that determined the effect of fatty acid supplementation on PMK-1 phosphorylation (Figure 5C). Supplementation with both GLA and SDA completely restored the level of phosphoryated PMK-1 in the *fat-3(wa22)* mutant to wild-type, indicating that both fatty acids are required to maintain basal PMK-1 activation, and thus the basal PMK-1-dependent MAP kinase immune function. Supplementation with ALA, AA or EPA, on the other hand, had no detectable effect on the levels of phosphorylated PMK-1 in the *fat-3(wa22)* mutant.

Supplementation with GLA and SDA also rescued the *fat-3(wa22)* response to oxidative stress, again functionally confirming the restoration of p38 MAP kinase activity with the supplementation of the two missing fatty acids (Figure S6). Supplementation with AA and EPA had no effect on the oxidative stress response, as expected from their lack on effect on PMK-1 phosphorylation or gene expression in the *fat-3(wa22)* mutant (Figure S6). We thus provide strong genetic evidence that two specific 18-carbon PUFAs, GLA and SDA play a vital role in maintaining basal activity of the p38 MAP kinase pathway and consequently influence both immune and stress responses in *C. elegans*. That the *fat-3* gene, through the synthesis of SDA and GLA, influences the basal expression of immune-specific genes, such as *spp-1*, *lys-2* and *lys-7*, and *lec-11*, further indicates that *fat-3* has a specific role in innate immunity, independent of its influences on oxidative stresses.

## Discussion

Using *C. elegans* mutants defective in PUFA biosynthesis, and detailed analysis of the Δ6-desaturase mutant *fat-3(wa22)*, we identified two 18-carbon PUFAs, GLA, an omega-6 fat, and SDA, an omega-3 fat, that play critical roles in basal immunity. Depletion of GLA and SDA resulted in disrupted basal activity of the p38 MAP kinase pathway and defective basal immune gene expression, leading to increased susceptibility to infection by *P. aeruginosa*. We also demonstrated that *fat-3* is required in the intestine, the site of *P. aeruginosa* infection, to protect *C. elegans* from pathogen-mediated death and to regulate the expression of immunity genes. The p38 MAP kinase pathway is required to protect *C. elegans* from infection and oxidative stress. Importantly, we showed that *fat-3*, through the synthesis of GLA and SDA, affects the expression of a subset of genes that are specifically required for immune function but not oxidative stress response. We further showed that loss of *fat-3* gene function does not result in a general loss of defense against stresses, and provided evidence that support an independent role for GLA and SDA in innate immunity.

Fatty acid desaturases have previously been shown to have important roles in innate immunity in mammals and plants. In mice, the stearoyl-Coenzyme A desaturase protein SCD1 is required for the production of immune effector molecules. SCD1 catalyses the Δ9 desaturation of 16- and 18-carbon saturated fatty acids into the monounsaturated palmitoleic (PLA, 16:1n9) and OA that are bactericidal against Gram-positive pathogens. Consequently, mice carrying loss of function *SCD1* mutations are defective in clearing skin infections by *Streptococcus pyogenes* and *Staphylococcus aureus* [54]. Whether these MUFAs are also involved in immune signaling remains to be investigated. In plants, mutants in the *Arabidopsis SSI2/FAB2* gene, which encodes a Δ9 desaturase, show enhanced resistance to bacterial and biotrophic oomycete fungal pathogens but increased susceptibility to a necrotrophic fungal pathogen [55]–[57]. The immune phenotypes of the *ssi2* mutant are due to low levels of OA, which leads to the constitutive activation of the salicylic acid-dependent immune pathway and repression of the jasmonic acid (JA)-dependent pathway by unknown mechanism(s) [55],[57],[58]. Disruption of another desaturase that catalyzes the conversion of LA to ALA, encoded by the *spr2* gene in tomato plants and *fad-7* and *fad-8* in *Arabidopsis*, also results in diminished JA signaling and a reduced response to wounding by insects and infection by fungal pathogens [59]–[61]. Similar to the lipid-dependent manipulation of immune signaling in plants, we showed for the first time that GLA and SDA, the products of FAT-3, an animal Δ6 desaturase, are required to maintain basal expression of immunity genes through their effect on the phosphorylation of a *C. elegans* p38 MAP kinase homolog, PMK-1.

In mammals, the omega-6 and omega-3 18-carbon PUFA families cannot be synthesized de novo. They must be produced from the dietary essential fatty acids, LA and ALA through a series of elongation and desaturation reactions. LA and ALA have relatively little pharmacologic action of their own; their effects derive largely from metabolic processing to more active end products. The human ortholog of the *C. elegans* FAT-3 enzyme, fatty acid desaturase 2 (FADS2) regulates production of GLA and SDA from their LA and ALA precursors [62]. This reaction is slow and can be further impaired by numerous factors, including aging, nutrient deficiencies, diabetes, hypertension, and life style factors, such as stress, smoking and excessive alcohol consumption [63],[64]. Thus, reduced dietary intake of LA and ALA, coupled with any of these conditions could lead to insufficient production of GLA and SDA in the body, potentially leading to compromised basal immunity analogous to the *C. elegans fat-3(wa22)* mutant. Reduced activity of FADS2 could also result in the decreased production of down-stream metabolites, such as the inflammatory mediators AA and EPA [64].

As noted above, we have provided several lines of evidence that the decreased ability of *fat-3* mutants to survive infection by *P. aeruginosa* is a consequence of diminished synthesis of GLA and SDA (Figure 2). By contrast, the 20-carbon PUFAs, AA and EPA appear to have minimal effects on the infection response to *P. aeruginosa*. Mutants deficient in different 20-carbon PUFAs, such as *elo-1(gk48)*, *elo-1(wa7)*, *fat-1(wa9)*, *fat-4(ok958)* and *fat-4(wa14)* show no defects in their response to infection (Figure 2A). Although supplementation with AA or EPA was sufficient to restore many of the additional defects displayed by the *fat-3(wa22)* mutant, neither of these PUFAs had any significant effects on the immune defects of *fat-3(wa22)*, as measured by survival on pathogen, expression of immune-specific genes and phosphorylation of PMK-1. In mammals, AA can be metabolized by cyclooxygenase, lipoxygenase and cytochrome P-450 (CYP) enzymes to produce important signaling molecules [3]. Since the *C. elegans* genome does not contain obvious orthologs of mammalian cyclooxygenases and lipoxygenases or of prostanoid and leukotriene receptors, a role for prostaglandin and leukotrienes in lipid signaling can be largely excluded. However, *C. elegans* shares with mammals the capacity to produce CYP-dependent eicosanoids. Recently, it was demonstrated that *C. elegans* contains microsomal monooxygenase systems, consisting of CYP-29A3 and CYP-33E2 cytochromes and an EMB-8 microsomal NADH-cytochrome c reductase that catalyze the epoxidation and hydroxylation of EPA and AA to specific sets of epoxy- and hydroxy-derivatives [65]. The ability of *C. elegans* to generate endogenous CYP-dependent eicosanoids could be blocked by inhibitors, such as adamantyl-3-dodecyl urea (ADU) developed against mammalian soluble epoxide hydrolases [65] suggesting that this component of eicosanoid metabolism may be conserved between *C. elegans* and mammals [66]. CYP-derived eicosanoids have been implicated in a variety of critical biological processes in humans, including homeostasis and inflammation [67]. Although our genetic analysis indicates that AA and EPA have no significant effect on the ability of *C. elegans* to survive infection by bacterial pathogens, we cannot not rule out other, as yet unidentified, roles for these 20-carbon fatty acids in the immune response.

CYP enzymes also play a role in the synthesis of oxylipins, oxygenated fatty acids synthesized from precursor PUFAs [68]. Oxylipins are typically derived from cis PUFAs, such as LA, ALA or AA [15], and act as signaling and effector molecules. Among the best known oxylipins are jasmonic acid and its derivatives that form vital signaling and effector molecules in plant immune responses [16]. In mammals, eicosanoids form one of the major groups of oxylipins, and are potent modulators of various physiological processes, including the regulation of inflammation [4]–[6]. Many oxylipins also show direct antimicrobial activities against bacteria, fungi and oomycetes [69]–[71]. A recent report indicated that in *Cyanobacteria*, GLA and SDA can be converted to oxylipins by CYP enzymes, but this process is not well characterized [72]. Little is known of oxylipin synthesis in *C. elegans*, but the presence of functional cytochrome P-450 enzymes leaves open the possibility that GLA and SDA could be processed into functional signaling molecules or immune effectors that directly influence the immune response. Future work will focus on determining if deficiency in CYP-derived eicosanoids or oxylipins could affect innate immune function in *C. elegans*.

The disruption of the FAT-3 Δ6-desaturase also resulted in altered immune gene expression and defective basal p38 MAP kinase activity (Figure 4). We demonstrate that this reduction in basal activity of p38 MAP kinase signaling and the concomitant increased susceptibility to both infection and oxidative stress, due to loss of *fat-3* function, are associated with GLA and SDA deficiencies. In *C. elegans*, the p38 MAP kinase is required for both the basal and induced expression of genes in response to infection [28] and functions through the activation of the p38 MAP kinase ortholog, PMK-1. Under normal growth conditions, this pathway is active as low levels of phosphorylated PMK-1 can be detected. We present the first evidence that the maintenance of PMK-1 basal activity requires GLA and SDA. Depletion of GLA and SDA in the *fat-3(wa22)* mutant significantly reduced the levels of phosphorylated PMK-1, without affecting the PMK-1 protein levels. This disruption further resulted in the altered basal expression of a number of immunity genes, as well as an increased susceptibility to oxidative stress. Despite retaining an intact response to infection in the absence of GLA and SDA, reduction in basal p38 MAP kinase signaling in the *fat-3* mutant was sufficient to cause increased susceptibility to both infection and oxidative stress, highlighting the vital importance of basal immunity. Previous research has similarly demonstrated the importance of this constitutive response in mammals and other invertebrates. In mammalian systems, beta-defensins form a major part of the constitutive immune response, and are continuously expressed in many epithelial tissues. Mice deficient in the production of the lung β-defensin-1 (mBD-1) showed defects in their ability to clear *H. influenzae* infections from the lung [73]. In this case, however, the mBD-1 mutant mice were defective in both the constitutive as well the inducible expression of the single effector molecules. Here, with the *fat-3* mutant, we demonstrate the essential requirement of a constitutive immune response pathway for survival against the pathogen, despite the presence of a functional inducible infection response in *C. elegans*. It would be additionally interesting to determine if GLA and SDA deficiencies in humans are also associated with reduced p38 MAP kinase activity and hypersensitivity to infection, and if these pathophysiological conditions could be restored through dietary supplementation of GLA and SDA.

The mechanism by which GLA and SDA affect the activity of p38 MAP kinase signaling and immune gene expression is currently unknown. GLA and SDA have a range of actions, and future work will be required to determine if their effects are direct or indirect. Lipids form a major constituent of cell membranes, and the effects of GLA and SDA may be associated with their influence on the physical properties of these membranes. The extent of membrane fatty acid unsaturation is known to influence membrane structure, fluidity and permeability [74]. Membrane fluidity is the extent of molecular disorder and molecular motion within the lipid bilayer [75]. This physical state of the membrane lipid can act directly to regulate membrane-bound proteins, such as receptor-associated protein kinases and ion channels, leading to alteration of gene expression [76],[77]. Thus, the effect of GLA and SDA depletion on reduced signaling through the p38 MAP kinase pathway may be linked to their effects on membrane fluidity, perhaps analogous to osmoregulation in yeast. When glucose was added to yeast medium to raise osmolarity, an associated reduction in membrane fluidity was observed [78]. When shifted to high osmolarity, yeast cells rapidly stimulate a MAP kinase cascade, the high-osmolarity glycerol (HOG) pathway, which orchestrates part of the transcriptional response [79]. Alternatively, the levels of SDA and GLA could affect lipid-protein interactions of membrane receptors and thus the intensity of signaling, analogous to the effects of OA on G protein coupled receptor (GPCR)-associated signaling. Addition of OA alters membrane structure and results in reduced G protein receptor activity in 3T3 cell derived membranes [80]. GPCRs are capable of activating MAPKs using an intricate signaling network [81]. It would be interesting to determine if depletion of GLA and SDA results in changes in membrane structure or fluidity that leads to reduced p38 MAP kinase signaling, through their effects on GPCRs or other membrane-associated signaling molecules.

Another important aspect of a cell membrane is its selective permeability, which plays a vital role in maintaining cell integrity and preventing entry of toxins [82]. Given that most pathogens secrete toxins and hydrolytic enzymes that can harm host cells, membrane permeability might affect the outcome of an infection. The *fat-3(wa22)* mutant is more sensitive to the detergent Triton X-100, potentially pointing to a defect in cell membrane permeability. However, the detergent sensitivity of the *fat-3(wa22)* mutant could be rescued without affecting its sensitivity to *P. aeruginosa* infection (Table 3, Figure 2C,D), indicating that the potentially altered cell membrane permeability of the *fat-3(wa22)* mutant does not impact immune function. Of note is that the *fat-3(wa22)* mutant also has a defective cuticle [42], which could account for, or partly influence, the detergent sensitivity of the *fat-3(wa22)* mutant, rather than a defect in membrane permeability.

Lipids perform a multitude of roles in the immune system, influencing both the innate and adaptive immune responses to infection. While their roles as inflammatory precursors is well known, studies have also identified lipid derived ligands that function through the mammalian Peroxisome Proliferators Activated Receptors (PPARs) to modulate the adaptive T cell response [83] and activate NK cells and dendritic cells [9],[10]. PPARs are a subset of nuclear hormone receptors (NHRs), a family of transcription factors activated by small lipophilic ligands that control a number of metabolic and systemic processes. In mammals, GLA is primarily converted to DGLA, a precursor of anti-inflammatory eicosanoids [64]. In keratinocytes, however, GLA treatment also results in the induction of COX-2 expression in a PPAR-γ-dependent manner. Addition of GLA results in the translocation of PPAR-γ to the nucleus and a consequent increase in COX-2 promoter activity and COX-2 protein levels in the cell [84]. This suggests a possible direct signaling role for GLA in regulating expression of the COX-2 gene, through PPAR-γ to mediate inflammatory immune responses. *C. elegans* has no known inflammatory response but does posses 284 putative NHRs [85], several of which affect the fat content [33] or the lipid metabolism of the worm [34],[86]. A number of these NHRs are differentially regulated in response to infection [27],[28], and reducing expression of *nhr-112*, by RNAi, results in increased sensitivity to infection by *P. aeruginosa* [27]. The interaction between lipid ligands, NHRs and the MAP kinase pathways has been explored previously in the context of the PPAR receptors in mammalian systems. In CD4^+^ T cells, unliganded PPARα suppresses p38 MAP kinase phosphorylation. Activation of PPARα by its lipid ligand relieves this restraint, allowing phosphorylation and activation of the MAP kinase pathway to induce cytokine production in these T cells [87]. Conversely, the p38 MAP kinase pathway has also been implicated in the control of PPARα activation and function. In vitro analysis shows that phosphorylation by p38 MAP kinase enhances activity of PPARα in cardiomyocytes [88], suggesting the possibility for similar complex interactions between the GLA and SDA PUFAs, NHRs and the p38 MAP kinase pathway in *C. elegans* innate immune function.

In summary, the demonstration that GLA and SDA are required for basal immunity adds to out understanding of the varied roles for lipids in immunity. Disrupting the synthesis of GLA and SDA leads to an increased sensitivity to infection, and the disrupted basal activity of the p38 MAP kinase pathway. Given that numerous conditions, including aging, diabetes, stress and smoking could lead to GLA and SDA deficiencies, it will be of interest to explore the roles for these PUFAs in other organisms, including humans.

## Materials and Methods

### Worm and Bacterial Strains

The strains *fat-2(wa17)*, *fat-3(wa22)*, *elo-1(wa7)*, *elo-1(gk48)*, *fat-1(wa9)*, *fat-4(ok958)*, *fat-4(wa14)*, *daf-16(mu86)*, *sma-6(wk7)*, *sek-1(km4)*, *pmk-1(km25)* and *pha-1(e2123)* were obtained from the *Caenorhabditis* Genome Center (CGC). The *fat-6(tm331)* strain was obtained from Dr. Shohei Mitani (National BioResource Project, Japan). The *fat-7(wa36)* and the *fat-6(tm331)*; *fat-7(wa36)* double mutant were gifts from Dr. Jennifer Watts (Washington State University). *fat-3(lg8101)* and the tissue specific rescue strains were gifts from Dr. Giovanni M Lesa (University College London). All strains were grown on nematode growth media (NGM) plates at 25°C and fed with the *E. coli* strain OP50-1 unless noted otherwise. For the assays described below, unless noted otherwise, all the worms were grown at 25°C on *E. coli* HT115 expressing the *pos*-1 RNAi construct to prevent the production of progeny [89]. Bacteria expressing dsRNA directed against *pos-1*, *sek-1*, *lys-7 and lec-11* were part of a *C. elegans* RNAi library expressed in *E. coli* strain HT115 (Geneservice, Cambridge, U.K.). Bacteria expressing dsRNA directed against *spp-1*, *lys-2*, *dct-17*, ZK6.11, *dod-19*, F49F1.1, F35E12.8, F08G5.6, *gst-38*, T01D3.6 were part of a *C. elegans* library expressed in *E. coli* strain HT115 (Open Biosystems, Huntsville, Alabama). All bacterial strains were cultured under standard conditions at 37°C.

### Fatty Acid Supplementation

ALA, AA and EPA supplements were obtained from NuChek Prep Inc., GLA was obtained from Sigma-Aldrich Co., while SDA was obtained from Cayman chemicals Co. Fatty acids were dissolved in 95% ethanol, and were added to a final concentration of 4 mM to *E. coli* HT115 carrying the *pos-1* RNAi construct and allowed to dry overnight in the dark. Wild-type animals fed *pos-1* RNAi bacteria supplemented with an equivalent amount of ethanol were used as controls. Worms were allowed to grow on the supplements from egg to one-day-old adults at a temperature of 25°C. For adult supplementation assays, young adult animals were placed on supplement plates for 48 hours before transfer. Supplementation, in each case, was verified by GC-MS, and collected worms were used for multiple assays.

### Survival Assays

Survival assays were performed as described [27]. Plates were scored every 12 hours, and worms that showed no response to touch were scored as dead. Worms that died due to desiccation or by bagging due to live progeny were excluded from the analysis. Statistical analyses were performed using a Kaplan-Meier non-parametric comparison and a Logrank test, using Statview (Version 5.0.1, SAS Institute Inc.). All assays were repeated a minimum of three times, with approximately 120 worms tested per condition in each assay.

### Gas Chromatography and Mass Spectometry (GC-MS)

Synchronized populations of several thousand adult worms were harvested at appropriate time points and washed with M9 buffer to remove excess bacteria. Worm pellets were treated with 3% H_2_SO_4_ in methanol and incubated at 80°C for 2 hours. Fatty acids were extracted as described previously [32] and analyzed by GC-MS using an HP 6890 gas chromatograph equipped with an HP-5MS column (30 m×0.25 mm×25 µm).

### Quantitative Real Time-PCR (qRT-PCR)

One-day-old adult animals were exposed to OP50-1, PA14 or PA14Δ*gacA* for 12 hours. For supplementation assays, worms were allowed to develop from embryos to young adults in the presence of fatty acids prior to analysis. RNA extraction and qRT-PCR were performed as previously described [27]. 25 µl reactions were performed using the iScript One-Step RT-PCR kit with SYBR green according to the manufacturer's instructions (BioRad Laboratories, Hercules, CA). Cycling threshold (Ct) values were normalized to mRNA levels of three primer pairs, pan actin (*act-1,3,4*), F44B9.5 and *ama-1*, which did not change with infection. Values and statistical analyses were calculated from normalized cycle threshold values prior to conversion to relative fold change.

### Colonization

Colonization assays were performed on slow killing plates using a GFP-expressing PA14 strain (PA14-GFP) [38] and the *pha-1(e2123)* temperature sensitive mutant strain, to avoid the presence of progeny on the assay plates at the restrictive temperature of 25°C. Adult worms were exposed to PA14-GFP for 24 hours and the intestinal bacterial load was determined under a fluorescence microscope. The degree of colonization was determined as follows: worms with a lumen completely packed with PA14-GFP were classified as fully colonized, worms that showed a limited presence of GFP in the intestine were classified as partially colonized and worms with no detectable GFP expression in the intestine were classified as having undetectable levels of colonization. A minimum of 2 independent experiments was performed with a total of 60 worms per sample per time point for each experiment. Statistical analyses were performed using Chi-square tests.

### Life Span

Life span assays were performed on NGM plates containing 0.1 mg/ml FUDR to prevent progeny from hatching [90]. Plates were seeded with concentrated OP50-1 and allowed to dry overnight. A synchronized population of L4 worms was placed onto the plates and scored every 24 hours. All strains were compared to the wild-type Bristol N2 strain and approximately 120 worms were used per strain per experiment. Statistical analyses were performed using a Kaplan-Meier non-parametric comparison and a Logrank test, using Statview (Version 5.0.1, SAS Institute Inc.).

### Immunoblot Analysis

Adult worm samples were washed with M9 and frozen for analysis. Animals were homogenized in M9 buffer and protein content was measured with a BCA Protein Assay Kit (Thermo Fisher Scientific Inc.) before loading. A phospho p38 specific monoclonal antibody (Cell Signaling Technology, Inc.), a p38 specific antibody (Cell Signaling Technology, Inc.) and an anti-actin antibody (Sigma-Aldrich Co.) were used at concentrations of 1∶1000, 1∶250 and 1∶250 respectively.

### Arsenic and Aldicarb Assays

Slow killing plates were coated with a final concentration of 3 mM sodium arsenite and allowed to dry overnight [53]. Plates were then seeded with *E. coli* OP50-1 and approximately 30 adult worms were placed on each plate, for a total of 120 worms per strain. Plates were scored every 12 hours and worms that showed no response to touch were counted as dead. Aldicarb (2-methyl-2-[methylthio]- propionaldehyde *O*-[methylcarbamoyl]oxime; Chem Services, West Chester, PA) stocks were dissolved in acetone and added to a final concentration 0.7 mM onto NGM plates [91]. Plates were allowed to dry overnight in the dark and then seeded with *E. coli* OP50-1. 30 adult worms were placed on each plate and monitored every 4–6 hours for paralysis, with approximately 120 worms used per strain/treatment for each experiment. Statistical analyses were performed using Kaplan-Meier non-parametric comparisons and Logrank tests, using Statview (Version 5.0.1, SAS Institute Inc.).

### Defecation and Movement Assays

Defecation assays were performed as described [42]. Defecation cycles were measured as the time between successive posterior body contractions over a period of five minutes. All assays were conducted in closed Petri dishes seeded with OP50-1, and a minimum of six adult animals was scored for per strain for each fatty acid treatment. Movement assays were performed as described [92] with M9 buffer in 96-well microtiter plates. A minimum of six animals was scored for total number of thrashes within a period of 2 minutes. One ‘thrash’ was defined as a change in the direction of bending at the mid-body.

### Heavy Metal and Detergent Stress Assays

Metal toxicity assays were performed as described [44]. Briefly, sets of three 1-day-old adults were allowed to lay eggs on plates containing either CdCl_2_ (30 µM) or CuSO_4_ (250 µM), for 3 hours. Adult worms were then removed, and the number of eggs on each plate was determined. After incubation at 25°C for 48 hours, the number of surviving adults was counted. The percentage of adults was determined as total number of adults divided by total number of eggs. For PUFA supplementation assays, fatty acids were added to the bacteria before seeding the plates. One-day-old adult animals were incubated in a solution of 1% Triton X-100 for one hour. Following removal from the detergent solution and one-hour recovery on NGM plates at 25°C, the number of survivors was determined. For supplementation assays, worms were grown in the presence of different fatty acids, before being placed in the detergent solution.

### Thermal Stress Assay

Heat and cold stress assays were carried out as described [93]. One-day-old adult animals were exposed to 0°C or 36°C for a period of 24 hours and 10 hours, respectively. Number of survivors was determined following one-hour recovery at 25°C. Significant differences in thermal tolerance were determined using a Student's *t*-test.

## Supporting Information

Figure S1 Fatty acid composition and virulence of OP50-1, PA14 and PA14Δ*gacA*. A. *P. aeruginosa* PA14 and PA14Δ*gacA* are similar in fatty acid content, but are highly divergent from *E. coli* OP50-1. GC-MS analysis was used to measure and identify species of fatty acids. Relative abundance of different fatty acid species are expressed as fraction of total fatty acids and depicted as mean±s.e.m. *, p≤0.001 between OP50-1 and PA14; Student's *t*-test. Abbreviations: PA, palmitic acid; PLA, palmitoleic acid; VA, vaccenic acid; MHA, cis-9,10 methylene hexadecanoic acid; MOA, cis-11,12 methylene octadecanoic acid. B. PA14Δ*gacA* is highly attenuated compared to the wild-type PA14. Survival analysis of wild-type worms exposed to OP50-1, PA14 and PA14Δ*gacA*. Graph depicts fraction of worms alive plotted as a function of time. *, p≤0.001 compared to OP50-1; Kaplan Meier non-parametric comparison and a Logrank test.
(0.5 MB TIF)

Figure S2 Exogenous PUFA supplementation restores levels of depleted fatty acids. (A) Wild-type worms simultaneously supplemented with GLA and SDA, or individually with AA or EPA. •, p≤0.001 between supplemented and untreated wild-type animals; Student's *t*-test. *fat-3(wa22)* supplemented with GLA and SDA (B), AA (C) or EPA (D). Relative lipid levels are expressed as percent of total LCFAs measured and represent mean±s.e.m. from three independent experiments. *, p≤0.001 compared to wild-type for *fat-3(wa22)*; #, p≤0.001 compared to wild-type for *fat-3* supplemented with GLA+SDA; Δ, p≤0.001 compared to wild-type for *fat-3* supplemented with AA; ▿, p≤0.001 compared to wild type for *fat-3* supplemented with EPA; Student's *t*-test. Abbreviations: AA, arachidonic acid; ALA, alpha-linolenic acid; DGLA, dihomo-γ-linolenic acid; EPA, eicosapentaenoic acid; GLA, gamma-linolenic acid; LA, linoleic acid; O3AA, ω-3 arachidonic acid; OA, oleic acid; PA, palmitic acid; PLA, palmitoleic acid; SA, stearic acid; VA, vaccenic acid.
(4.4 MB TIF)

Figure S3 Exogenous PUFA supplementation has no effect on survival of wild-type and *elo-1(gk48)* animals. A, B. Exogenous PUFA supplementation has no effect on survival of wild-type animals against PA14 infection. Survival analysis of wild-type animals supplemented with 18-carbon (A) or 20-carbon (B) PUFAs. C. GLA supplementation in adult *fat-3(wa22)* animals partially rescued survival. Adult *fat-3(wa22)* animals were supplemented with ALA, GLA or SDA for a period of 48 hours before exposure to PA14. D. 18-carbon PUFA supplementation has no effect on PA14 resistant *elo-1(gk48)* animals. All graphs depict fraction of worms alive as a function of time. *, p≤0.001 compared to wild-type; Kaplan Meier non-parametric comparison and a Logrank test. Abbreviations: AA, arachidonic acid; ALA, alpha-linolenic acid; EPA, eicosapentaenoic acid; GLA, gamma-linolenic acid; SDA, stearidonic acid.
(3.7 MB TIF)

Figure S4 Misregulated expression of infection-response genes in infected *fat-3(wa22)* animals. A. Increased rate of bacterial colonization in *lec-11* and F49F1.1 RNAi-treated animals challenged with PA14-GFP compared to wild-type (p≤0.001; Chi-square test). Animals grown on RNAi bacteria were exposed to PA14-GFP and monitored for extent of intestinal colonization after 24 h. Figure depicts percent of worms in each colonization category, for each RNAi gene target. B. Expression levels of infection response genes significantly altered in PA14 infected *fat-3(wa22)* animals (p≤0.05; Student's *t*-test). Data are depicted as mean±s.e.m., and represent fold expression relative to wild-type animals infected with PA14.
(1.6 MB TIF)

Figure S5 Intestinal *fat-3* expression restores basal expression of infection response genes. A. qRT-PCR analysis of infection-response gene expression in *fat-3(lg8101)* transgenic animals expressing the *fat-3* gene under its endogenous promoter. B-D. Effect of intestinal (B), neuronal (C) or muscle (D) specific *fat-3* expression on basal infection-response gene expression. Graphs depict mean±s.e.m. and represent fold difference in gene expression relative to wild-type animals on OP50-1, with wild-type set to 1. #, p>0.05 for the *fat-3; [Pfat-3::fat-3]* strain. ▵, •, ▿, p>0.05, respectively for the intestine- (*Pelt-2::fat-3*), muscle- (*Pmyo-3::fat-3*) and neurons- (*Punc-119::fat-3*) specific *fat-3* rescue strains compared to wild-type; Student's *t*-test. Horizontal line under each graph identifies genes that are specifically required for survival against PA14 infection (see Table 4).
(3.6 MB TIF)

Figure S6 Exogenous PUFA supplementation restores *fat-3* response to oxidative stress. Supplementation with GLA and SDA rescues *fat-3* susceptibility to oxidative stress. Untreated and PUFA supplemented animals were placed on plates containing 3 mM arsenic, and survival was determined every 12 h. Graph depicts fraction of worms alive plotted as a function of time. *, p≤0.001 compared to wild-type; Kaplan Meier non-parametric comparison and a Logrank test. Abbreviations: AA, arachidonic acid; EPA, eicosapentaenoic acid; GLA, gamma-linolenic acid; SDA, stearidonic acid.
(2.2 MB TIF)

Table S1 Analysis of 50 stress- and infection-response genes. q-RT PCR analysis of stress- and infection-response gene expression in uninfected and *P. aeruginosa*-infected wild-type, *fat-3(wa22)*, *sek-1(km4)*, *pmk-1(km25)*, *daf-16(mu86)* and *sma-6(wk7)* animals. Data are depicted as Ct values relative to wild-type±s.e.m. (Ct_wild-type – mutant_) and represent average values of three independent experiments. Student's *t*-test was used to determine significant differences in gene expression.
(0.04 MB XLS)

Table S2 Basal expression of infection response genes in two different *fat-3* alleles. Comparative qRT-PCR analysis of expression of 12 infection and stress-response genes between wild-type, *fat-3(wa22)* and *fat-3(lg8101)*. Data are depicted as Ct values relative to wild-type±s.e.m. (Ct_wild-type–mutant_) and represent average values of three independent experiments. * p>0.05 compared wild-type; Student's *t*-test.
(0.05 MB DOC)
