# Supplementary figures and images for: Gamma-Linolenic and Stearidonic Acids Are Required for Basal Immunity in Caenorhabditis elegans through Their Effects on p38 MAP Kinase Activity
Source: PLoS Genet. 2008 Nov 21;4(11):e1000273. doi: 10.1371/journal.pgen.1000273 (PMC2581601; doi:10.1371/journal.pgen.1000273)

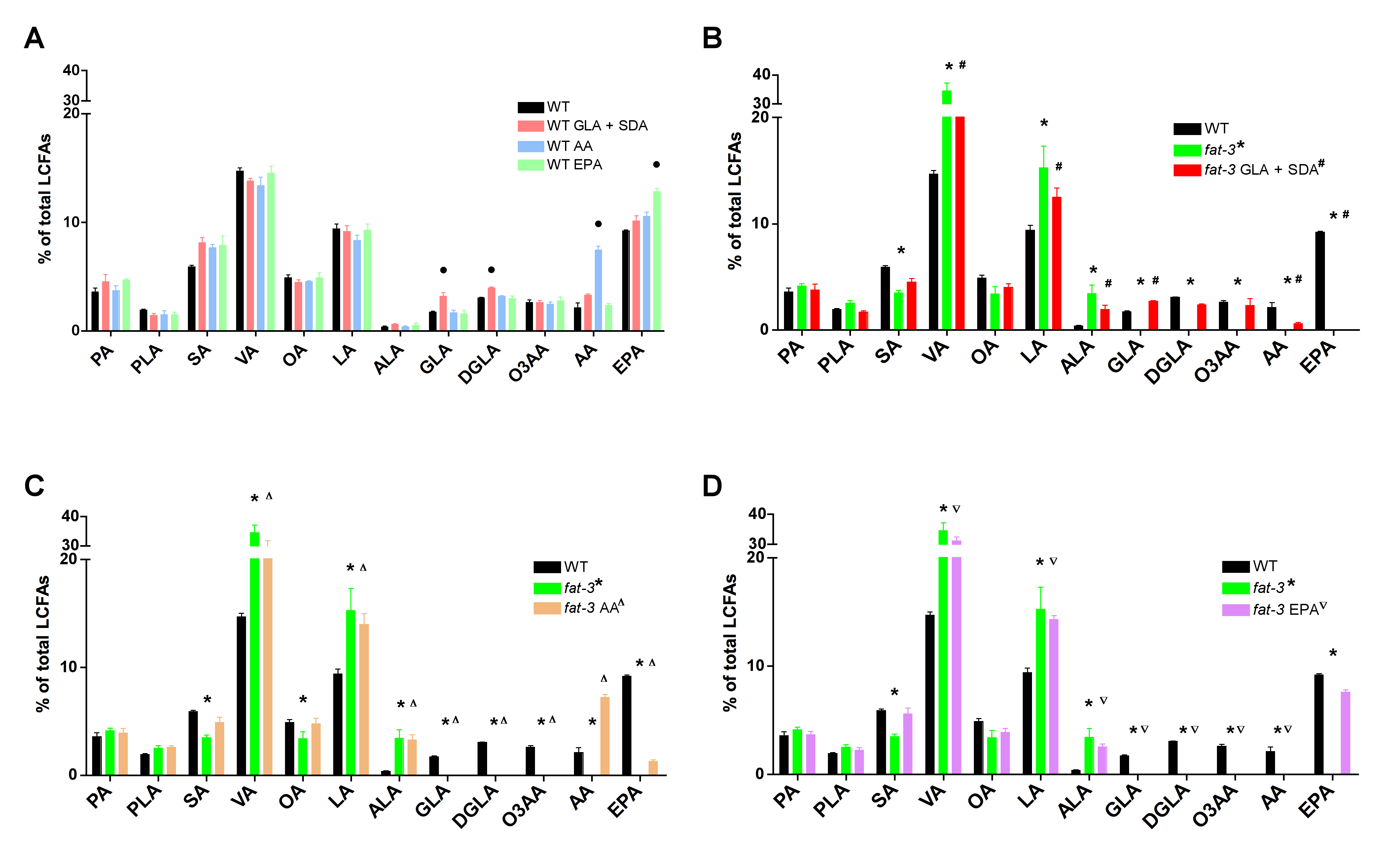

Supplement: Figure S2 — Exogenous PUFA supplementation restores levels of depleted fatty acids. (A) Wild-type worms simultaneously supplemented with GLA and SDA, or individually with AA or EPA. •, p≤0.001 between supplemented and untreated wild-type animals; Student's t-test. fat-3(wa22) supplemented with GLA and SDA (B), AA (C) or EPA (D). Relative lipid levels are expressed as percent of total LCFAs measured and represent mean±s.e.m. from three independent experiments. *, p≤0.001 compared to wild-type for fat-3(wa22); #, p≤0.001 compared to wild-type for fat-3 supplemented with GLA+SDA; Δ, p≤0.001 compared to wild-type for fat-3 supplemented with AA; ▿, p≤0.001 compared to wild type for fat-3 supplemented with EPA; Student's t-test. Abbreviations: AA, arachidonic acid; ALA, alpha-linolenic acid; DGLA, dihomo-γ-linolenic acid; EPA, eicosapentaenoic acid; GLA, gamma-linolenic acid; LA, linoleic acid; O3AA, ω-3 arachidonic acid; OA, oleic acid; PA, palmitic acid; PLA, palmitoleic acid; SA, stearic acid; VA, vaccenic acid. (4.4 MB TIF) [file pgen.1000273.s002.tif]

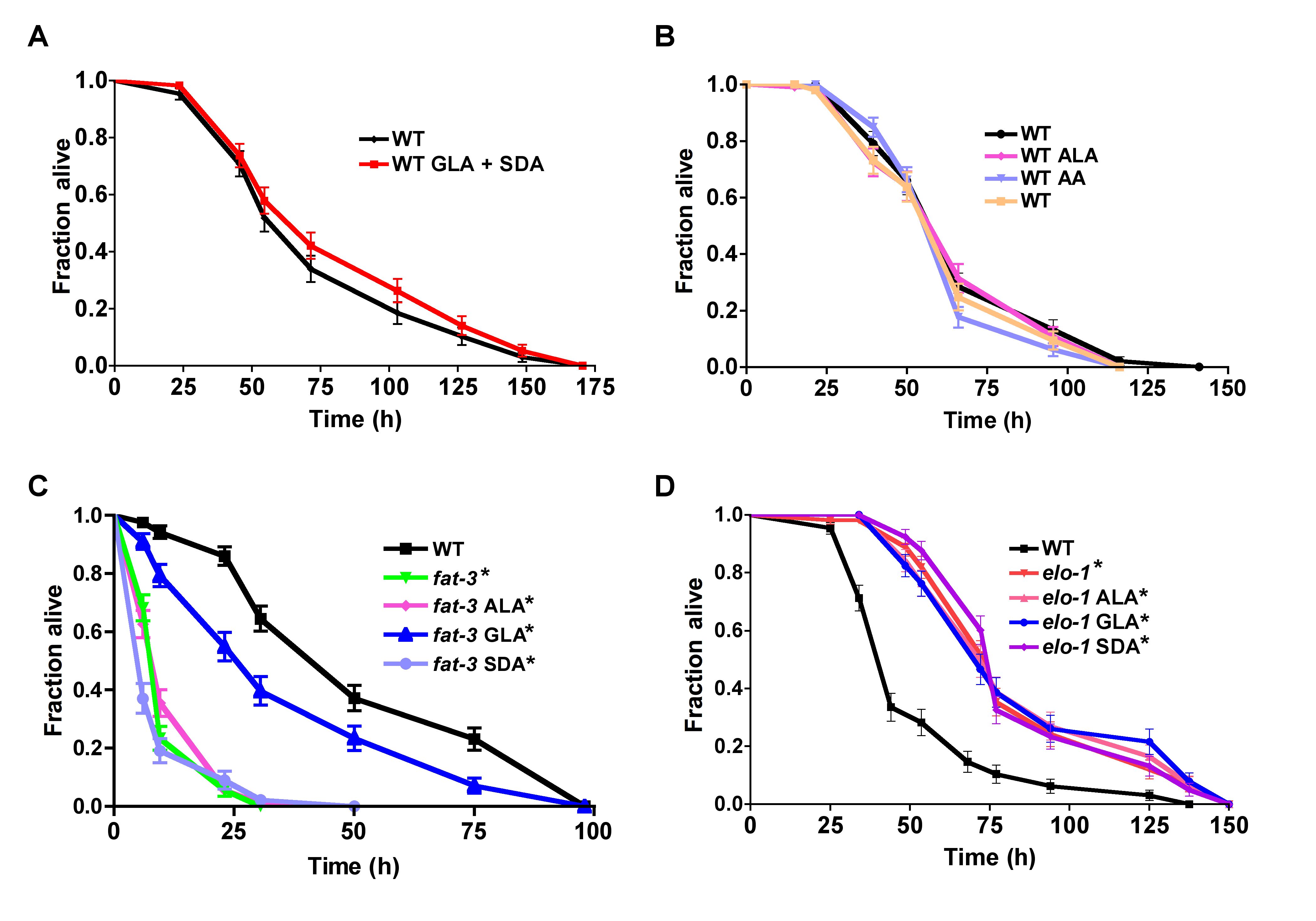

Supplement: Figure S3 — Exogenous PUFA supplementation has no effect on survival of wild-type and elo-1(gk48) animals. A, B. Exogenous PUFA supplementation has no effect on survival of wild-type animals against PA14 infection. Survival analysis of wild-type animals supplemented with 18-carbon (A) or 20-carbon (B) PUFAs. C. GLA supplementation in adult fat-3(wa22) animals partially rescued survival. Adult fat-3(wa22) animals were supplemented with ALA, GLA or SDA for a period of 48 hours before exposure to PA14. D. 18-carbon PUFA supplementation has no effect on PA14 resistant elo-1(gk48) animals. All graphs depict fraction of worms alive as a function of time. *, p≤0.001 compared to wild-type; Kaplan Meier non-parametric comparison and a Logrank test. Abbreviations: AA, arachidonic acid; ALA, alpha-linolenic acid; EPA, eicosapentaenoic acid; GLA, gamma-linolenic acid; SDA, stearidonic acid. (3.7 MB TIF) [file pgen.1000273.s003.tif]

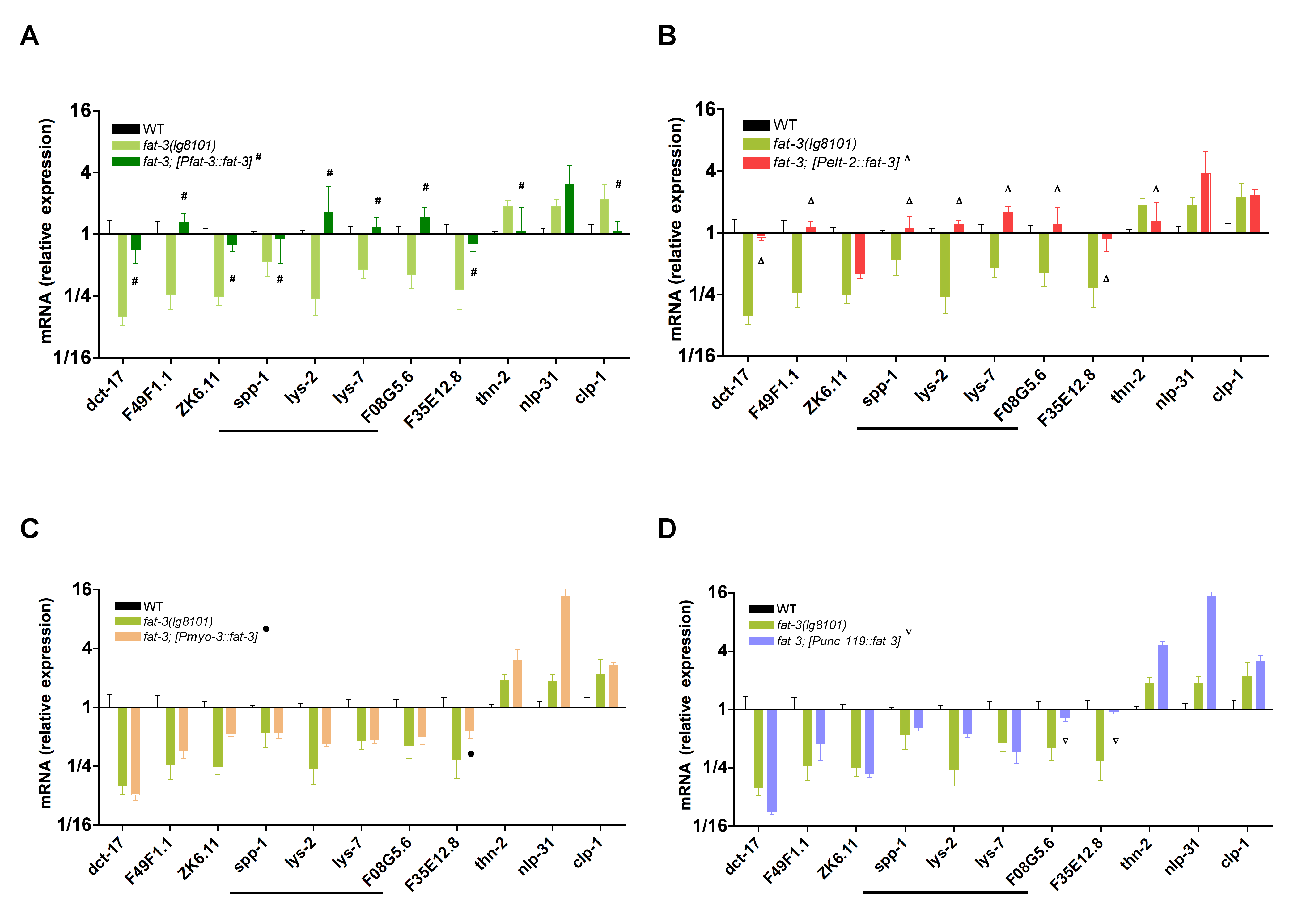

Supplement: Figure S5 — Intestinal fat-3 expression restores basal expression of infection response genes. A. qRT-PCR analysis of infection-response gene expression in fat-3(lg8101) transgenic animals expressing the fat-3 gene under its endogenous promoter. B-D. Effect of intestinal (B), neuronal (C) or muscle (D) specific fat-3 expression on basal infection-response gene expression. Graphs depict mean±s.e.m. and represent fold difference in gene expression relative to wild-type animals on OP50-1, with wild-type set to 1. #, p>0.05 for the fat-3; [Pfat-3::fat-3] strain. ▵, •, ▿, p>0.05, respectively for the intestine- (Pelt-2::fat-3), muscle- (Pmyo-3::fat-3) and neurons- (Punc-119::fat-3) specific fat-3 rescue strains compared to wild-type; Student's t-test. Horizontal line under each graph identifies genes that are specifically required for survival against PA14 infection (see Table 4). (3.6 MB TIF) [file pgen.1000273.s005.tif]

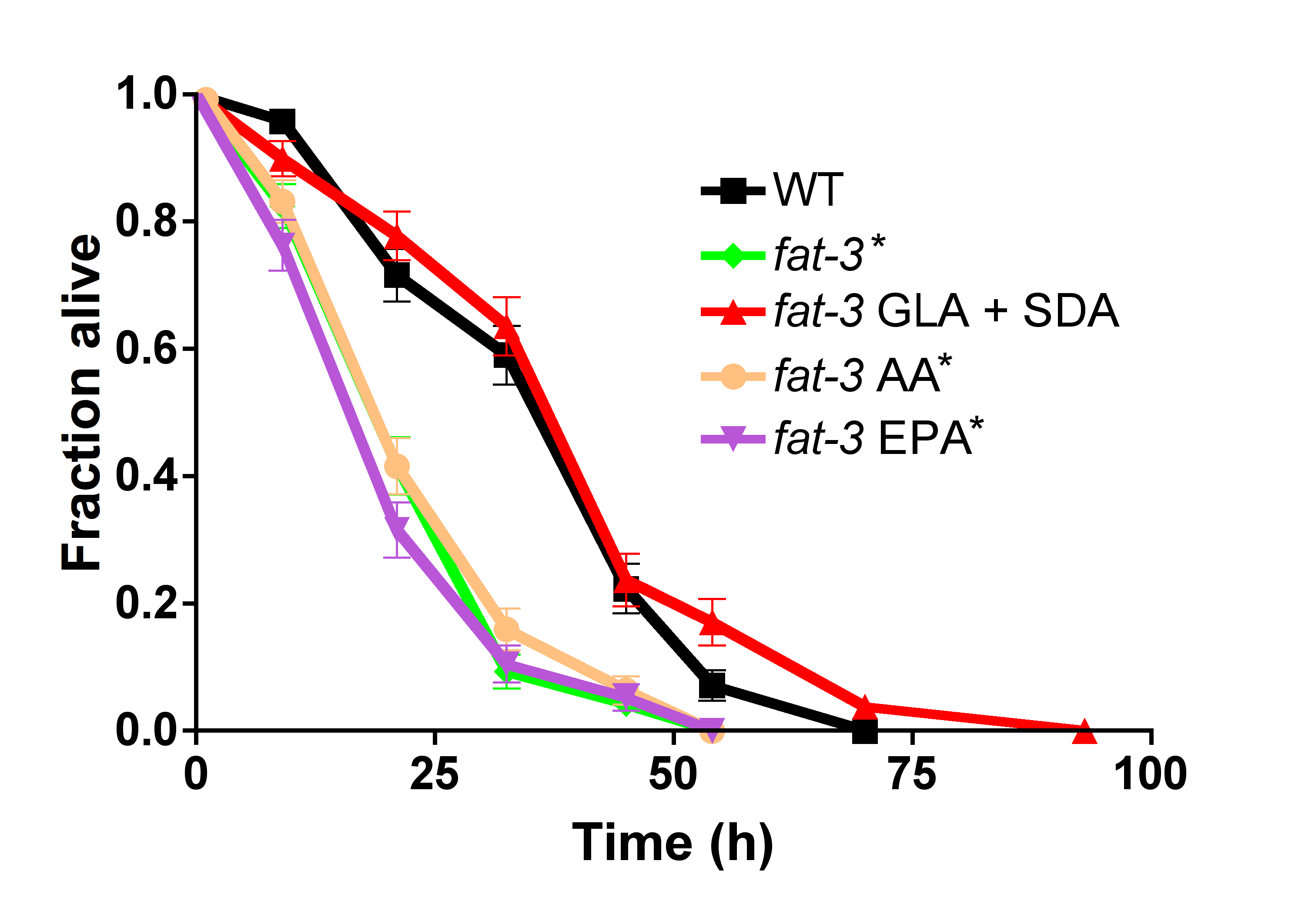

Supplement: Figure S6 — Exogenous PUFA supplementation restores fat-3 response to oxidative stress. Supplementation with GLA and SDA rescues fat-3 susceptibility to oxidative stress. Untreated and PUFA supplemented animals were placed on plates containing 3 mM arsenic, and survival was determined every 12 h. Graph depicts fraction of worms alive plotted as a function of time. *, p≤0.001 compared to wild-type; Kaplan Meier non-parametric comparison and a Logrank test. Abbreviations: AA, arachidonic acid; EPA, eicosapentaenoic acid; GLA, gamma-linolenic acid; SDA, stearidonic acid. (2.2 MB TIF) [file pgen.1000273.s006.tif]
