# Supplementary material for: Gamma-Linolenic and Stearidonic Acids Are Required for Basal Immunity in Caenorhabditis elegans through Their Effects on p38 MAP Kinase Activity
Source: PLoS Genet. 2008 Nov 21;4(11):e1000273. doi: 10.1371/journal.pgen.1000273 (PMC2581601; doi:10.1371/journal.pgen.1000273)
Supplement: Table S2 — Basal expression of infection response genes in two different fat-3 alleles. Comparative qRT-PCR analysis of expression of 12 infection and stress-response genes between wild-type, fat-3(wa22) and fat-3(lg8101). Data are depicted as Ct values relative to wild-type±s.e.m. (Ctwild-type–mutant) and represent average values of three independent experiments. * p>0.05 compared wild-type; Student's t-test. (0.05 MB DOC) [file pgen.1000273.s008.doc]

| **Gene** | **Sequence Id** | **Gene expression relative to wild-type on OP50-1**  **(Ct_WT – mutant_ ± s.e.m.)** | | |
| --- | --- | --- | --- | --- |
|  |  | **wild-type** | ***fat-3(wa22)*** | ***fat-3(lg8101)*** |
| *dct-17* | F35E12.7 | 0 ± 0.45 | -2.72 ± 0.09 | -2.67 ± 0.29 |
| F49F1.1 | F49F1.1 | 0 ± 0.41 | -2.93 ± 0.58 | -1.93 ± 0.50 |
| ZK6.11 | ZK6.11 | 0 ± 0.18 | -1.03 ± 0.26 | -2.00 ± 0.26 |
| *spp-1* | T07C4.4 | 0 ± 0.09 | -0.96 ± 0.23 | -0.89 ± 0.50 |
| *lys-2* | Y22F5A.5 | 0 ± 0.13 | -1.27 ± 0.28 | -2.06 ± 0.55 |
| *lys-7* | C02A12.4 | 0 ± 0.26 | -1.57 ± 0.52 | -1.13 ± 0.31 |
| F08G5.6 | F08G5.6 | 0 ± 0.25 | -2.54 ± 0.28 | -1.32 ± 0.45 |
| *lec-11* | F38A5.3 | 0 ± 0.06 | -1.43 ± 0.49 | -0.41 ± 0.16* |
| F35E12.8 | F35E12.8 | 0 ± 0.32 | -0.94 ± 0.15 | -1.76 ± 0.66 |
| *thn-2* | F28D1.5 | 0 ± 0.10 | 1.53 ± 0.09 | 0.92 ± 0.21 |
| *nlp-31* | B0213.6 | 0 ± 0.20 | 0.93 ± 0.12 | 0.89 ± 0.23 |
| *clp-1* | C06G4.2 | 0 ± 0.32 | 1.43 ± 0.35 | 1.13 ± 0.48 |

Comparative q-RT PCR analysis of expression of 12 infection-response genes between wild-type, *fat-3(wa22)* and *fat-3(lg8101)*. Data are depicted as Ct values relative to wild-type ± SEM (Ct_wild-type – mutant_) and represent average values of three independent experiments. Student’s t-test was used to determine significant differences in gene expression. * p > 0.05 compared to wild-type; Student’s *t*-test.
